# Stress‐Induced Domain Wall Motion in a Ferroelastic Mn^3+^ Spin Crossover Complex

**DOI:** 10.1002/anie.202003041

**Published:** 2020-06-08

**Authors:** Vibe B. Jakobsen, Elzbieta Trzop, Laurence C. Gavin, Emiel Dobbelaar, Shalinee Chikara, Xiaxin Ding, Kane Esien, Helge Müller‐Bunz, Solveig Felton, Vivien S. Zapf, Eric Collet, Michael A. Carpenter, Grace G. Morgan

**Affiliations:** ^1^ School of Chemistry University College Dublin Belfield Dublin 4 Ireland; ^2^ Univ Rennes CNRS, IPR (Institut de Physique de Rennes)—UMR 6251 35000 Rennes France; ^3^ Department of Physics Auburn University Auburn AL 36849 USA; ^4^ National High Magnetic Field Laboratory Los Alamos National Laboratory Los Alamos NM 87545 USA; ^5^ Centre for Nanostructured Media School of Mathematics and Physics Queen's University of Belfast Belfast BT7 1NN, Northern Ireland UK; ^6^ Department of Earth Sciences University of Cambridge Downing Street Cambridge CB2 3EQ UK; ^7^ Current address: Technische Universität Kaiserslautern Kaiserslautern Germany; ^8^ Current address: National High Magnetic Field Lab at Florida State University Tallahassee FL USA; ^9^ Current address: Idaho National Laboratory Idaho Falls ID USA

**Keywords:** domain wall, ferroelastic materials, manganese(III), spin crossover, structural phase transition

## Abstract

Domain wall motion is detected for the first time during the transition to a ferroelastic and spin state ordered phase of a spin crossover complex. Single‐crystal X‐ray diffraction and resonant ultrasound spectroscopy (RUS) revealed two distinct symmetry‐breaking phase transitions in the mononuclear Mn^3+^ compound [Mn(3,5‐diBr‐sal_2_(323))]BPh_4_, 1. The first at 250 K, involves the space group change *Cc*→*Pc* and is thermodynamically continuous, while the second, *Pc*→*P*1 at 85 K, is discontinuous and related to spin crossover and spin state ordering. Stress‐induced domain wall mobility was interpreted on the basis of a steep increase in acoustic loss immediately below the the *Pc*‐*P*1 transition

## Graphical abstract

**Stresses and strains**: Domain wall motion is detected for the first time in a spin crossover crystal. Mobility of ferroelastic domain walls was interpreted on the basis of a steep increase in acoustic loss below a first order transition. Spin state ordering and domain formation is associated with collapse of the Jahn–Teller distortion on switching from the spin quintet to spin triplet form of a mononuclear Mn^3+^ complex.

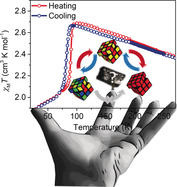

## Introduction

Domain walls (DWs) in ferroic materials—ferromagnets, ferroelectrics, ferroelastics—represent the regions where there is a change in order parameter.^1^ The dimensions, mobility, and internal structure of domain walls continue to yield useful functionality such as magnetic racetrack memory,^2^ in which the supersonic motion^3^ of magnetic DWs is driven by spin‐polarized currents. In the last decade, work on ferroelectric oxides has unexpectedly revealed that electrical conductivity,^4^ or even superconductivity,^5^ is possible within ferroelectric DWs, despite the fact that ferroelectrics should be good insulators. Thus, far from being an inert barrier between functional ordered regions, the DW in both ferromagnets and ferroelectrics is instead recognized as a functional entity in itself, and is being investigated for applications in which “the wall is the device”.^1^ In this context, it is of interest to examine other types of ordered materials to probe the nature of DW structure and to look for new functionality.

Whilst most reports on ferroic properties focus on inorganic oxides, molecular systems also offer a rich playground for structural and electronic ordering. For example, in molecular crystals both intramolecular and intermolecular degrees of freedom can be modulated to induce changes in either local point‐group and/or global translational symmetry, as has been demonstrated in organic ferroelectric materials.^6^ The vibronic phenomenon of thermal spin state switching^7^ is also well known to cause significant structural reorganization in both small‐molecule transition‐metal complexes^8^ and solid‐state oxides.^9^ In spin crossover (SCO) materials, the switching is usually strongly coupled to structural degrees of freedom, with local bond‐length changes of up to 0.2 Å in each metal–donor distance due to depopulation/population of anti‐bonding orbitals during the electron pairing/unpairing process. These local distortions at the molecular scale propagate macroscopically through elastic coupling, resulting in macroscopic changes in lattice parameters.^10^ The variety of SCO phenomena can be understood in terms of the evolution of the totally symmetric HS fraction order parameter, *γ*, which may couple to a symmetry‐breaking order parameter driving spin state ordering, *η*, or to volume and shear strains.^11^ Such coupling, in turn, can give rise to large anomalies in elastic properties.^12^ In some SCO crystals, this drives cooperative phase transitions to produce multiple structural phases with spin state ordering over a temperature gradient.^13^ Such ordering phenomena have been the focus of sustained experimental^14^ and theoretical^11^, ^15^ investigations over the last decade but little is known about the DW architecture in the ordered phases, as in most systems studied so far, spin state ordering results in antiphase boundaries. Herein, we report magnetic, structural, and elastic properties of a new Mn^3+^ SCO complex, [Mn(3,5‐diBr‐sal_2_(323))]BPh_4_, **1**, and show that the ferroelastic DWs in one of two spin state ordered phases are mobile in response to shear stress. The DWs detected in complex **1** are distinct from the high‐spin/low‐spin (HS/LS) phase boundary, which develops in crystalline SCO materials across a thermal gradient, and in which spatiotemporal effects can be very effectively followed by optical microscopy.^16^ Such examples of an isostructural phase transition between LS and HS phases do not correspond to DW formation, rather to a phase boundary. In the isostructural case both HS and LS phases have the same symmetry, so the symmetry‐breaking order parameter is 0 and the HS/LS interface is not a DW. In contrast, in the case of complex **1**, the spin state and ferroelastic order parameters are both coupled with strain, making it inevitable that the DWs will contain local variations in spin state, thus realizing a new class of DW architecture.

## Results and Discussion

**Figure 1:**
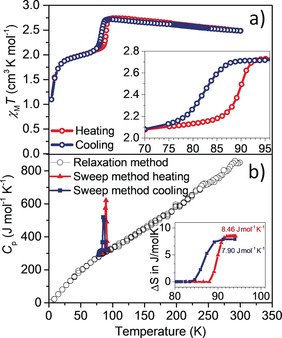
a) Plot of *χ*_M_ *T* versus *T* for complex **1** in cooling (blue curve) and heating (red curve) modes between 4 and 300 K measured at 0.1 T. Inset: 8 K wide hysteretic transition. b) Heat capacity, *Cp*, versus *T* of a single crystal of complex **1** measured by two methods, the relaxation method (black circles) and the temperature sweep method (red line, warming; blue line, cooling). The temperature sweep method is sensitive to sharp changes such as first order phase transitions, whereas the relaxation method more accurately determines the magnitude of the heat capacity where it is smoothly varying with temperature. Inset: Entropy change Δ*S* determined from integration of the peak in the heat capacity: 7.90 J mol^−1^ K^−1^ on cooling and 8.46 J mol^−1^ K^−1^ on heating.

**Scheme 1:**
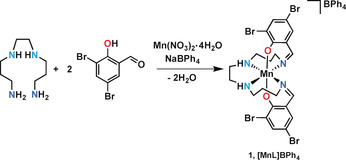
Synthesis of complex **1**, [Mn(3,5‐diBr‐sal)_2_323]BPh_4_.

[Mn(3,5‐diBr‐sal_2_(323))]BPh_4_, **1**, belongs to the [Mn(R‐sal_2_(323))]^+^ series of Schiff base complexes, many of which exhibit thermal SCO or stabilization of the rare *S*=1 state.14f, 17 Dark red crystals of complex **1** were prepared in a one‐pot synthesis, Scheme 1, and magnetic susceptibility in heating and cooling modes over the temperature range 4–300 K was recorded on a SQUID magnetometer in an applied field of 0.1 T, Figure 1 a and Figure S1.

Plots of *χ*_M_ *T* versus *T*, Figure 1 a, indicate that complex **1** is in its spin quintet form at room temperature. A 9.3 % increase in *χ*_M_ *T* was observed on cooling from 300 K (2.49 cm^3^ mol^−1^ K) to 95 K (2.72 cm^3^ mol^−1^ K), whereupon an abrupt drop to a *χ*_M_ *T* value of 2.1 cm^3^ mol^−1^ K was observed with a T1/2 ↓ value of 82 K, Figure S2. This represents a 50:50 ratio of spin quintet and triplet forms. A further decrease on cooling below circa 20 K is observed, which is attributed to zero‐field splitting. On heating, an abrupt and hysteretic transition was observed with T1/2 ↑=90 K.

Thus, we identify a first‐order phase transition related to spin state switching centred at 86 K with a thermal hysteresis window of 8 K. The width of the hysteresis is of the same order of magnitude as reported for other Mn^3+^ SCO complexes with an N_4_O_2_^2−^ ligand donor set.14f, 17c The transition at circa 86 K was accompanied by a change in entropy of circa 8 J mol^−1^ K^−1^, obtained by integration of the peak from heat capacity measurements, Figure 1 b. Such a large entropy change suggests that a significant component of the thermodynamic driving force is configurational, which involves both structural and electronic reorganizations accompanying the SCO behaviour. No influence of magnetic field on the heating branch was observed and only a slight upward shift in the cooling branch by 1.5 K was observed when applying fields of 1 T and 5 T, Figure S3.

Resonant ultrasound spectroscopy (RUS) revealed that three structural phases emerge over the temperature interval of the SCO, including a structural state that contains ferroelastic twin domains (see below). Single‐crystal diffraction was used to elucidate the structure in each phase and the full transition sequence is *Cc*→*Pc*→*P*1.

**Figure 2:**
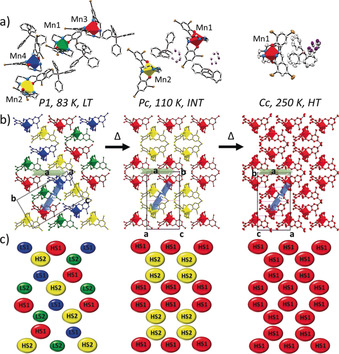
Perspective view of the LT *P*1 (83 K), INT *Pc* (110 K) and HT *Cc* (250 K) structures of complex **1**.^34^ a) Representation of the asymmetric unit with distorted [MnN_4_O_2_]^+^ units shown as polyhedra with colour coding: HS1 (red), HS2 (yellow), LS1 (blue) and LS2 (green). b) The same structures displayed in a layered crystal packing arrangement, showing the relationships between the conventional unit cells of each. c) Simplified representation of the three structures in terms of the Mn atoms alone, with the same colour coding as in (a,b). Atoms are shown at 50 % atomic probability distributions for ellipsoids. BPh_4_^−^ anion in (b), (c) and hydrogen atoms are omitted for clarity.

At room temperature, complex **1** crystallises in the monoclinic polar space group *Cc* and data in this high temperature (HT) phase was collected at 293 K and at 250 K, Table S4. The asymmetric unit comprises one full occupancy [Mn^III^L]^+^ cation, which is chelated by a hexadentate *trans*‐N_4_O_2_‐ligand, Figure 2 a and Figure S5. By symmetry, the global polarization in the *Cc* space group lies on the *(a,c)* plane. The geometry around the Mn^3+^ centre can be described as a distorted octahedron even though the bonds involve different atoms, Mn−N_amine/imine_ and Mn−O_phen_, with bond lengths in the equatorial plane showing significant elongation, Figure S4 and Table S5. This is consistent with population of the $d_{x^{2}-y^{2}}$ orbital of the anti‐bonding e_g_* orbitals in the Jahn–Teller distorted *S*=2 state. The asymmetric unit also contains one disordered BPh_4_^−^ counteranion, Figure 2 a and Figures S5 and S6.

Single‐crystal X‐ray diffraction data collected at different temperatures revealed a slight change in the slope of lattice parameter variations below circa 250 K and an abrupt change below circa 90 K, Figures S30 and S31. Collection of a full data set at intermediate temperatures (INT), 150 K and 110 K, confirmed a change in symmetry by the appearance of reflections not obeying the reflection conditions for the *Cc* cell, Figures S32 and S33. The structure was refined under monoclinic polar space group *Pc* and the asymmetric unit in this INT phase comprises two unique [Mn^III^L]^+^ cations, both in the HS *S*=2 state. There are increases in Mn−N_imine_ and Mn−N_amine_ bond lengths in both sites compared with the single *S*=2 site in the structures at 293 K and 250 K. The asymmetric unit in the INT phase also contains two unique disordered BPh_4_^−^ counteranions, Figure 2 a and Figures S7 and S8.

The lattice parameters show a steep decrease in *a* and increases in *b* and *c* below the transition at circa 90 K, Figures S30 and S31. A different set of superstructure reflections, characteristic of the loss of the *c* glide plane, was observed in a full data set collected at 83 K and 25 K, the low‐temperature (LT) phase, indicating that it has a different symmetry from the higher temperature phases. The symmetry decrease requires refining the structure in the space group *P*1, which is chiral and polar. The unit cell contains four independent [Mn^III^L]^+^ cations and four BPh_4_^−^ counteranions, the latter now in a fully ordered configuration, Figure 2 a and Figures S9 and S10. Two of the four Mn^3+^ cations are in the *S*=1 state and two are in the *S*=2 state. There is no indication of a geometric Jahn–Teller effect in the *S*=1 Mn^3+^ cations. The Mn−N_imine_ and Mn−N_amine_ bond lengths in all the measured structures are similar to those of other related Mn^3+^ *S*=1 and *S*=2 complexes, Table S5.14f, 17

Values of ΣMn, *Φ*, and *ζ*,^18^ which define the degree of octahedral distortion in relation to spin state changes, are also higher, as expected, for the *S*=2 Mn^3+^ cations in the HT and INT phases (293 K, 250 K, 150 K, 110 K) than for the LT *S*=1 Mn^3+^ cations observed at 83 K and 25 K, Table S6.

Only weak hydrogen‐bonding interactions between the Mn^3+^ complex cation(s) and the BPh_4_^−^ counteranion(s) were found in HT (250 K), INT (110 K) and LT (83 K) phases, Figures S20–S25. A full Hirshfeld surface analysis, mapped over *d*_norm_, of complex **1**, Figures S26–S28, shows that the three main contributions to the intermolecular interactions are H⋅⋅⋅H, H⋅⋅⋅Br and H⋅⋅⋅C with an increase in H⋅⋅⋅Br and a decrease in H⋅⋅⋅H interactions in the LT phase compared to the INT and HT phase, Figure S29. The slight changes in intermolecular interactions may generate a critical elastic energy, which directly affects the spin state causing the hysteretic response between the INT and LT phases. The spin state distribution across the three phases is summarized in Tables S5 and S6 and in the structure diagrams in Figure 2 b,c and Figures S12–S19 in which the striped spin state order of the Mn^3+^ complex cations in the low‐temperature regime is apparent.

The thermal evolution of complex **1** is unambiguously associated with the two phase transitions in the sequence *Cc*→*Pc*→*P*1. Given the group–subgroup relationship between the *Cc* and *Pc* space groups, which have the same translation symmetry (*a*,*b*,*c*),^19^ the *Cc*→*Pc* transition is allowed to be second order. The related order parameter, *q*, describing the associated structural order, has the symmetry of irreducible representation (irrep) at the border of the Brillouin zone, *Y_1_*. This is not the case for the transition between the *Pc* and *P*1 phases because there is no group–subgroup relationship; some translation symmetry operator exists in the *Pc* phase and not in the *P*1 phase and vice versa, Figure 2. Such a reconstructive phase transition must be first order.

**Figure 3:**
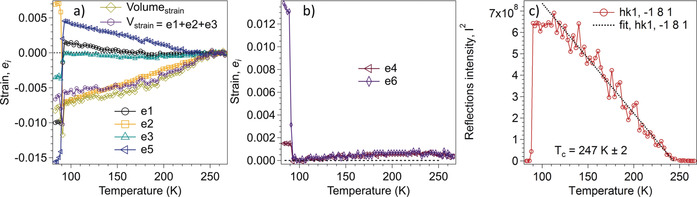
a, b) Temperature dependence of strain components for the *Pc* and *P*1 structures, as defined with respect to the parent *Cc* structure. All the strains vary continuously through the *Cc*→*Pc* transition at circa 247 K and discontinuously through the *Pc*→*P*1 transition at circa 90 K. c) Variations of the square of the intensity, *I*_k_, of the superstructure reflection with *hk*1=−181, which appears in diffraction patterns from the *Pc* phase. The data show *I*_k_^2^∝(*T*_c_−*T*), within experimental uncertainty, and *T*_c_=247±2 K.

Lattice distortions associated with structural, magnetic, electronic or any other phase transition between structures that have a group–subgroup relationship depend formally on coupling between a thermodynamic order parameter, *q*, and components of a second rank strain tensor, [*e*_i_].^20^ The lowest order coupling terms permitted by symmetry are *λ_i_* *e_i_* *q* ^2^, *i*=1,2,3,5, and *λ_i_* *e_i_* ^2^ *q* ^2^, *i*=4,6, for *Cc*→*Pc*. Coupling of the form *λ_i_* *e_i_* *q* ^2^ gives *e_i_*∝*q* ^2^.^20^ Strain variations calculated from lattice parameter data in Figure S36 reveal unambiguously that *q* ^2^ for the *Cc*→*Pc* transition varies continuously through the transition temperature, *T*_c_, and has a non‐linear dependence on temperature in the stability field of the *Pc* structure, Figure 3 a.

The precise form of the non‐linearity is highly sensitive to the choice of baseline for the reference states. An alternative approach is to follow by X‐ray diffraction the temperature dependence of the intensity *I_k_* of superlattice reflections associated with the *Cc*→*Pc* transition, corresponding to Bragg peaks (*hkl*) with *h*+*k* odd. Figure 3 c shows that below *T_c_* it appears that the fit of *I*_k_^2^ has linear dependence so *I*_k_^2^∝(*T*_c_−*T*) and, hence the order parameter scales as, *q* ^4^∝(*T*_c_−*T*), indicating that the transition is close to being Landau tricritical in character with *T*_c_=247±2 K. By way of contrast, the *Pc*→*P*1 transition is very obviously first order, with a large discontinuity at circa 90 K, Figure 3 a–c.

**Figure 4:**
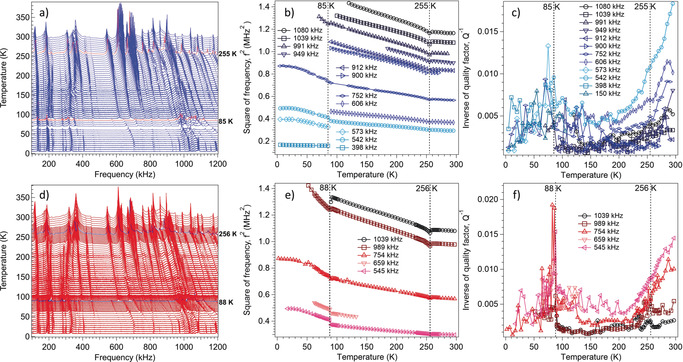
a,d) RUS spectra as a function of frequency for a single crystal of complex **1**, stacked up the *y*‐axis in proportion to the temperature at which they were collected. The *y*‐axis is really amplitude in volts but has been relabelled as temperature. Spectra were collected during (a) cooling and (d) heating between circa 295 K and circa 4 K. The highlighted red lines (cooling) and blue lines (heating) indicate the expected location of the transitions at circa 255 K and circa 85 K. b,e) *f* ^2^ and c,f) *Q* ^−1^ data from fitting of selected resonances with an asymmetric Lorentzian function, showing the continuous structural phase transition at circa 255 K and the discontinuous transition at circa 85 K.

The two phase transitions are also evident in variations in elastic properties obtained by RUS from a small single crystal. This technique is commonly used to investigate phase transitions,^21^ and has been used once previously for a non‐symmetry‐breaking SCO material.^22^ The square of the frequencies, *f*, of mechanical resonance peaks of a single crystal scales with different combinations of elastic moduli. The peak widths at half maximum height provide a measure of acoustic dissipation in the form of the inverse mechanical quality factor, *Q* ^−1^. A stack of spectra collected from a single crystal during cooling from 295 to 4 K, Figure 4 a, revealed a small shift in the frequency trends of all resonance peaks below circa 250 K. There was then a more marked shift in resonance frequencies below circa 85 K. The widths of individual peaks also increased abruptly below circa 85 K. On heating back up to room temperature from 7 K, Figure 4 d, the resonance peaks returned to the same positions as in the cooling sequence, confirming that the crystal survived the two phase transitions without cracking.

It is well understood that changes in the elastic constants of single crystals at phase transitions depend primarily on the form and strength of the coupling between the driving order parameter and strain.^23^ Coupling of the form *λ_i_* *e_i_* *q* ^2^ is expected to give rise to discontinuous softening as the crystal is cooled through the transition temperature of a classical displacive transition, with the magnitude of the effect depending sensitively on *λ_i_* ^2^. However, the *Cc*→*Pc* transition is marked by a slight minimum in *f* ^2^ followed by an increase in the slope of the stiffening trend with falling temperature. The *Pc*→*P*1 transition is marked by a small discontinuity and a larger increase in slope of the stiffening trend, Figure 4 b,e. Such stiffening occurs either when the values of *λ_i_* are negligibly small, which is not the case here, or when the time required for relaxation of the order parameter in response to a strain induced by some external stress is short in comparison with the timescale of the applied stress. Changes in the elastic constants during the *Pc*→*P*1 transition can be attributed to the partial spin state conversion due to the stronger bonding nature of the LS state.

**Figure 5:**
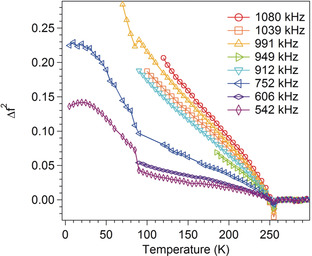
Variations of changes in *f* ^2^ with respect to values for the *Cc* structure obtained by extrapolation of linear fits to data collected above 255 K.

As shown in Figure 5, variations of *f* ^2^ for the *Pc* phase with respect to values extrapolated linearly from the stability field of the *Cc* structure, expressed as Δ*f* ^2^, have nonlinear form similar to variations of the strains, that is, *e_i_*∝Δ*f* ^2^∝*q* ^2^. This confirms that relaxation of the order parameter requires times greater than circa 10^−6^ s, given that the observed resonance frequencies are circa 10^5^–10^6^ Hz. The pattern of acoustic loss also adds insights into the nature of the phase transitions. A normal expectation for displacive transitions is that *Q* ^−1^ values, Figure 4 c,f, are low in the high‐symmetry phase, with the possibility of a peak at the transition point marking a critical slowing down of the atomic motions responsible for the transitions, and high in the low‐symmetry phase if there is a loss mechanism involving a transformation microstructure such as ferroelastic twin walls.^21^

Instead, the steep reduction in values of *Q* ^−1^ as the *Cc*→*Pc* transition point is approached from above is more reminiscent of the magnetic ordering transition in YMnO_3_,^24^ as well as structural transitions involving hydrogen bonding in a metal–organic framework^25^ and in the mineral lawsonite.^26^ In each of these cases, the transitions were interpreted as having a significant component of order–disorder character, and this was confirmed by calorimetric measurements in the case of lawsonite.^27^ Dynamical clustering of ordered regions ahead of the transition contributes to relatively high acoustic losses by coupling with local strains and this disappears below the transition when the ordering is static.

By way of contrast with the *Cc*→*Pc* transition, there is a steep increase in *Q* ^−1^ immediately below the *Pc*→*P*1 transition, as would be typical of a ferroelastic transition in which the loss is due to mobility of ferroelastic twin walls in response to an applied stress. Typical examples are (Ca,Sr)TiO_3_ and Sr(Zr,Ti)O_3_ perovskites at temperatures below the cubic–tetragonal transition.^28^ The RUS evidence of acoustic loss is thus that crystals with *P*1 symmetry contain ferroelastic twins even when they developed by a first‐order transition from the *Pc* structure, Figure S16, and that the twin walls are at least partially mobile under the influence of external stress. Given that there is coupling between the ferroelastic and spin state order parameters, it is inevitable that these domain walls will contain local variations in the degree of spin state order that also must respond to the external stress.

As described in the introduction, the discovery of mobile ferroelastic DWs in complex **1** is quite distinct from the motion of the HS/LS boundary in isostructural single‐crystal SCO samples^16^ and instead represents a new phenomenon. It will now be important to establish a method to determine the velocity of DW motion in ferroelastic SCO systems, which will enable meaningful comparison with their ferromagnetic and ferroelectric counterparts. In these latter materials there are marked differences between the magnetic‐field induced supersonic speeds of 750–1000 m s^−1^ achievable in ferromagnetic thin films^29^ and nanowires,^30^ and the much slower motion of ferroelectric DWs, in which velocities are also much more sensitive to sample preparation and orientation.^31^ Internal DW structure is also typically complex in ferroic materials; in ferromagnets, in which the magnitude of the quantized spins cannot change across the wall, the magnetization is inverted by chiral out‐of‐plane (Néel) or in‐plane (Bloch) rotation of the spins.^1^ In contrast, ferroelectric DWs are Ising‐like as the non‐quantized polarization axis can vary in size and gradually reverse its sign.^1^ It will therefore be of interest to further probe the internal structure of ferroelastic DWs in SCO crystals, nanomaterials7a and films^32^ using advanced imaging techniques suitable for different physical scales.^8^

## Conclusion

In summary, we have demonstrated formation of mobile ferroelastic twin walls in a Mn^3+^ SCO crystal with strong coupling between spin state and elastic order parameters. The spin quintet form of Mn^3+^ SCO compounds exhibits a pronounced Jahn–Teller effect, which can be easily injected into or removed from the lattice by changing the spin state via thermal or other perturbations. This large change in structural distortion is likely to have contributed to the considerable elastic strain in the *Pc*→*P*1 transition. As spin state switching results in a change in both magnetization, through a change in the overall spin state of the transition metal ion, and large atomic displacements, such compounds are ideal for magnetoelectric applications. These include, for example, data storage devices with an electrical input and magnetic read‐out, which would avoid the problems of reading ferroelectric random access memory (FeRAM).^33^ In the case of complex **1**, all three structural phases are polar and therefore potentially ferroelectric. Thus, it is of interest to explore these aspects and to investigate the potential for ferroelectric ordering and DW conductivity in our ongoing studies on this and related materials.

## Conflict of interest

The authors declare no conflict of interest.

## Supporting information

As a service to our authors and readers, this journal provides supporting information supplied by the authors. Such materials are peer reviewed and may be re‐organized for online delivery, but are not copy‐edited or typeset. Technical support issues arising from supporting information (other than missing files) should be addressed to the authors.

Supplementary

Supplementary

Supplementary

Supplementary
