# Supplementary material for: Stress‐Induced Domain Wall Motion in a Ferroelastic Mn3+ Spin Crossover Complex
Source: Angew Chem Int Ed Engl. 2020 Jun 8;59(32):13305–12. doi: 10.1002/anie.202003041 (PMC7496919; doi:10.1002/anie.202003041)
Supplement: Supplementary file 1 — Supplementary [file ANIE-59-13305-s001.pdf]

Angewandte Chemie
Eine Zeitschrift der Gesellschaft Deutscher Chemiker

Supporting Information

Stress-Induced Domain Wall Motion in a Ferroelastic Mn^{3+} Spin Crossover Complex

Vibe B. Jakobsen, Elzbieta Trzop, Laurence C. Gavin, Emiel Dobbelaar, Shalinee Chikara, Xiaxin Ding, Kane Esien, Helge Müller-Bunz, Solveig Felton, Vivien S. Zapf, Eric Collet, Michael A. Carpenter,* and Grace G. Morgan**

anie_202003041_sm_miscellaneous_information.pdf
anie_202003041_sm_Video_1.mkv
anie_202003041_sm_Video_2.avi
anie_202003041_sm_Video_3.avi

SUPPORTING INFORMATION

Table of contents

1. Experimental procedures

1.1 Synthesis S2

1.2 Magnetometry by superconducting quantum interference device (SQUID) S2

1.3 Heat capacity S2

1.4 Single crystal X-ray diffraction S2

1.5 Resonant ultrasound spectroscopy S3

2. Magnetometry measurements

3. Single crystal X-ray diffraction

3.1 Crystal and structure refinement data S9

3.2 Crystal structures and packing of complex **1** S15

3.3 Specific intermolecular interactions of complex **1** S24

3.4 Hirshfeld surface mapping S27

3.5 Variable temperature single crystal X-ray diffraction S31

3.6 Reconstructed planes and superstructure reflections S33

3.7 Twin domains S36

4. Resonant ultrasound spectroscopy

4.1 Strain analysis from unit cell parameters S37

4.2 Correlation of transition points between different measurements S39

5. Author contributions

6. References

1. Experimental procedures

1.1 Synthesis

Materials: All chemicals were purchased from Sigma Aldrich. All other reagents were purchased from standard sources and were used as received.

Synthesis of [Mn^{III}(3,5-diBr-sal_2(323))]BPh_4, complex **1**

N,N-bis(aminopropyl)ethylenediamine (0.55 mmol, 0.0962 g) dissolved in 1:1 CH_3CN/EtOH (15 mL), was mixed with 3,5-dibromosalicylaldehyde (1.02 mmol, 0.2842 g) dissolved in 1:1 CH_3CN:EtOH (15 mL) forming a yellow solution. This was allowed to stir for 15 min. Solid manganese(II) nitrate tetrahydrate (0.54 mmol, 0.1351 g) was added to a solution of sodium tetraphenylborate (0.51 mmol, 0.1751 g) in 1:1 CH_3CN:EtOH (10 mL). The two solutions were then mixed, yielding a dark brown solution of the Mn(III) complex resulting from air oxidation of the initial Mn(II) salt. Dark red crystals of complex **1** suitable quality for X-ray diffraction were formed within a day upon slow evaporation of the solvent (337.6 mg, 62%). Large single crystals were obtained by recrystallization from 1:1 DMF/CH_3CN over several weeks. Elemental analysis calcd. (found) (%) for $C_{46}H_{44}BN_4O_2MnBr_4$: C, 51.62 (51.44); H, 4.14 (4.04); N, 5.23 (5.16). IR (FT-ATR diamond anvil) v/cm^{-1} = 3236(w), 1674(w), 1621(m), 1576(m), 1515(w), 1452(w), 1421(m), 1374(m), 1244(m), 1183(w), 1164(w), 1073(m), 985(m), 842(m), 734(m), 708(s), 608(s), 487(m), 469(m).

1.2 Magnetometry by superconducting quantum interference device (SQUID)

The magnetic susceptibility of complex **1** was measured on a Quantum Design MPMS XL SQUID magnetometer. The magnetic susceptibility was measured twice on the same polycrystalline packed sample. The first measurement was performed to study the spin crossover (SCO) profile and field dependence of the hysteresis loop. The second measurement was more detailed in both regions of the structural phase transitions and was achieved by reducing the temperature intervals from 5 K to 1 K for every data acquisition point. Both measurements were performed over a temperature range of 4 to 300 K in both heating and cooling modes for verification of the hysteretic behaviour of complex **1**.

The first measurement of the magnetic susceptibility was taken in 5 K intervals from 300 K to 4 K in both heating and cooling modes. Field dependence of the hysteresis window centered at 86 K was investigated from 110 K to 70 K at 0.1 T, 1 T and 5 T in cooling and heating modes.

The second measurement was recorded in 5 K intervals from 300 K to 250 K. Intervals of 1 K were used in the temperature range 190-250 K and 5 K intervals were used in the range 95-190 K. Between 75-95 K, 1 K intervals were used for a more precise measurement of the changes close to the first order phase transition below 90 K. 5 K intervals were used in the range 4-75 K. The same temperature intervals were used in heating and cooling modes.

Diamagnetic corrections were calculated for complex **1** using Pascal's constants and applied to all data. The calculated $\chi_M T$ vs T data is listed in Table S1 and S2-S3 for the two sets of measurements respectively.

1.3 Heat capacity

Heat capacity was measured on a single crystal of complex **1** in a 14 Tesla Quantum Design Physical Properties Measurement System (PPMS) in the range 2-250 K.[1] Two methods were employed: the standard relaxation method was used to determine the heat capacity in absolute units over the whole temperature range. The second method, known as the temperature sweep, or long pulse method, was used to determine the heat capacity and the latent heat in the immediate vicinity of the spin crossover transition. In this method, a large heat pulse was applied and the temperature change vs time was monitored while crossing the phase transition.[1] The mass of the single crystal of complex **1** was 1 mg ± 0.03.

1.4 Single crystal X-ray diffraction

Single crystal X-ray diffraction (SCXRD) data of complex **1** was collected first at 100 K on a suitable single crystal using an Oxford Supernova X-ray diffractometer (Oxford Instruments, Oxford, United Kingdom) with a micro-source, using a Mo-Kα (λ = 0.71073 Å) radiation, and fitted with an ATLAS detector at the School of Chemistry, University College Dublin, Ireland. A second series of experiments was subsequently completed at Institut de Physique de Rennes, Université de Rennes 1, France, where SCXRD data on a new crystal of complex **1** was collected on an Agilent Technologies SuperNova Single Source X-ray diffractometer with a micro-source, using Cu-Kα (λ = 1.54184 Å) radiation, and fitted with an EosS2 detector. Here, data were taken at 250 K, 150 K, 110 K, 83 K and 25 K for structure determination and, with a change of temperature from 250 K to 83 K (every 2-3° step with 200K/h cooling/heating

rate) to monitor the variations of cell parameters and Bragg peaks intensities. Nitrogen flow 800Plus series cryostat from Oxford Cryosystems was used for the measurements within the temperature range from 266 K to 83 K. Helium flow Oxford Diffraction Helijet Cryostream was used only for the measurement at 25 K. We used the same crystal for both nitrogen and helium environment experiments.

Complex **1** crystallises in the non-centrosymmetric polar space group *Cc* (data at 293 K and 250 K). Below 200 K due to the thermal transition complex **1** undergoes a symmetry change in the crystal to the non-centrosymmetric polar space group *Pc* (data at 150 K and 110 K). An additional thermal transition appears below 95 K to the non-centrosymmetric chiral and polar space group *P1* (data at 83 K and 25 K) and is accompanied by the formation of domains.

CrysAlisPRO[2] software package from Rigaku Oxford Diffraction was used for all data collections and data processing (indexing, integration and reduction). Full datasets were collected assuming that the Friedel pairs are not equivalent. This allowed for the sufficient data coverage for non-centrosymmetric crystallographic systems. Data scaling as well absorption corrections were applied during the final data processing. All structures were solved by dual direct methods with *Shel*XT[3] and refined by full matrix least-squares on F^2 using *Shel*XL[4] in *OLEX^2 GUI*[5]. The structures are all polar. All non-hydrogen atoms were refined anisotropically; H-atoms were constrained by geometry. When applicable, restrains: DFIX, DANG, SADI, SIMU and ISOR, were applied for occupational disorder of BPh_4^- anions or Br atom sites. Figure S11 shows the degree of occupational disorder in the BPh_4^- anions at various temperatures. For data at 83 K and 25 K, the twin refinement was applied due to formation of domains below 95 K upon thermal phase transition.

Data collection and refinement details are given in Table S4; the bond length and bond angle details are provided in Table S5. The evolution of the bond lengths from 293 K - 83 K is shown in Figure S4 for all three different HT, INT and LT structural phases. Perspective views of the asymmetric unit of the structures measured at 293 K, 250 K, 150 K, 110 K, 83 K and 25 K are provided in Figures S5-S10 with 50% atomic probability distributions for ellipsoids. The disorder of BPh_4^- anions in the unit cell for each full data collection is highlighted in Figure S11. Figures S30-S31 show changes of the unit cell parameters with temperature on heating and cooling. Precession images in (hk0) and (0kl) planes were calculated for 250 K, 110 K and 83 K datasets of **1** and are shown respectively in Figure S32-S33.

CCDC-1971455 (293 K), CCDC-1971456 (250 K), CCDC-1971457 (150 K), CCDC-1971458 (110 K), CCDC-1971459 (83 K) and CCDC-1971460 (25 K) contain the crystal data collection and refinement parameters details for this paper, which can be obtained free of charge via www.ccdc.cam.ac.uk/conts/retrieving.html (or from the Cambridge Crystallographic Data Centre, 12 Union Road, Cambridge CB2 1EZ, UK; fax: (+44) 1223-336-033; or deposit@ccdc.ca.ac.uk).

1.5 Resonant ultrasound spectroscopy

Resonant ultrasound spectroscopy (RUS) measurements were performed using a custom built design as previously described.[6] A single crystal of complex **1** (<1 mm in all directions) was mounted lightly between two piezoelectric transducers in the RUS head which was attached to the end of a stick for lowering into an Oxford Instruments Teslatron PT cryostat. Spectra were collected in the frequency range 100 to 1200 kHz at 5 K intervals from 295 K to 5 K in cooling mode. In heating mode, spectra were collected in the frequency range 100 to 1200 kHz at 5 K intervals from 7 K to 57 K. From 62 K to 102 K, a 2 K interval was employed and from 102 K to 232 K, a 5 K interval was used. From 232 K to 272 K a 2 K interval was used, and from 272 K to 297 K, a 5 K interval was employed. A 15 min settle time at each temperature step was set to allow for thermal equilibration before spectra were collected. All spectra were transferred to the software package IgorPro for analysis. A plot of the spectra (amplitude as a function of frequency) stacked as a function of temperature in small temperature intervals provides an easy way to observe and follow the trends of resonant frequencies as they evolve with temperature. Fits based on an asymmetric Lorentzian function give the values of peak frequencies, f, and the peak widths at half height, Δf, for selected resonance peaks. f^2 scales with the combination of elastic constants which determines each resonance mode. The inverse mechanical quality factor, Q^{-1} was taken as $Q^{-1} = (f/\Delta f)^{-1}$ which is a measure of acoustic loss.

2. Magnetometry measurements

Table S1. Magnetic data for polycrystalline sample of complex **1** in cooling and heating mode, first measurement

**Cooling mode**		**Heating Mode**	
**Temperature (K)**	**$\chi_M T$ (cm^3 K mol^{-1})**	**Temperature (K)**	**$\chi_M T$ (cm^3 K mol^{-1})**
300.02	2.5950	4.00	0.9337
294.21	2.5974	10.03	1.4056
287.30	2.6097	16.05	1.6223
281.27	2.6057	22.12	1.7366
275.26	2.6108	28.16	1.8057
269.23	2.6130	34.20	1.8513
263.20	2.6182	40.24	1.8884
257.16	2.6240	46.30	1.9211
251.14	2.6281	52.35	1.9507
245.11	2.6339	58.42	1.9808
239.05	2.6367	64.49	2.0099
233.03	2.6398	70.55	2.0412
226.99	2.6430	76.61	2.0862
221.01	2.6504	82.68	2.1323
214.94	2.6532	88.73	2.3662
208.89	2.6570	94.81	2.7035
202.86	2.6624	100.84	2.7101
196.84	2.6671	106.91	2.7121
190.80	2.6706	112.98	2.7117
184.78	2.6781	119.07	2.7130
178.74	2.6813	125.12	2.7098
172.71	2.6864	131.19	2.7086
166.69	2.6881	137.17	2.7041
160.66	2.6922	143.28	2.7004
154.60	2.6944	149.31	2.7000
148.60	2.6994	155.36	2.6960
142.55	2.7023	161.44	2.6926
136.52	2.7080	167.50	2.6905
130.49	2.7101	173.55	2.6865
124.47	2.7126	179.61	2.6799
118.45	2.7160	185.67	2.6718
112.45	2.7165	191.72	2.6722
106.48	2.7162	197.77	2.6700
100.47	2.7192	203.82	2.6640
94.56	2.7135	209.86	2.6578
88.63	2.6808	215.92	2.6517
82.62	2.3527	221.99	2.6520
76.58	2.1223	228.03	2.6477
70.52	2.0495	234.09	2.6399
64.51	2.0132	240.13	2.6342
58.45	1.9822	246.18	2.6305
52.38	1.9521	252.23	2.6269
46.30	1.9205	258.28	2.6238
40.24	1.8868	264.35	2.6157
34.19	1.8498	270.37	2.6151
28.15	1.8064	276.42	2.6099
22.12	1.7389	282.47	2.6087
16.08	1.6225	288.49	2.6074
10.07	1.4034	294.52	2.6028
4.00	0.9336	300.57	2.6029

Table S2. Magnetic data for polycrystalline sample of complex **1** in cooling mode, second measurement

Cooling mode					
**Temperature (K)**	**$\chi_M T$ (cm^3 K mol^{-1})**	**Temperature (K)**	**$\chi_M T$ (cm^3 K mol^{-1})**	**Temperature (K)**	**$\chi_M T$ (cm^3 K mol^{-1})**
300.0471	2.4845	218.7341	2.5795	129.6693	2.6790
294.1975	2.4911	217.7340	2.5795	124.6971	2.6858
289.5586	2.4960	216.7296	2.5811	119.7176	2.6943
284.4989	2.4997	215.7408	2.5829	114.7664	2.6988
279.4918	2.5027	214.7351	2.5843	109.8292	2.7068
274.4885	2.5082	213.7439	2.5851	104.8799	2.7111
269.4899	2.5134	212.7466	2.5867	99.9073	2.7164
264.4887	2.5190	211.7334	2.5879	94.9684	2.7168
259.5025	2.5244	210.7426	2.5889	93.8889	2.7149
254.5157	2.5285	209.7421	2.5902	92.8120	2.7138
249.5533	2.5355	208.7429	2.5903	91.8214	2.7133
248.7221	2.5426	207.7495	2.5919	90.8130	2.7122
247.7454	2.5433	206.7449	2.5932	89.8154	2.7095
246.7489	2.5458	205.7518	2.5964	88.8246	2.7026
245.7439	2.5456	204.7427	2.5976	87.8103	2.6926
244.7323	2.5460	203.7490	2.5996	86.8273	2.6685
243.7490	2.5471	202.7460	2.6015	85.8200	2.6315
242.7291	2.5480	201.7614	2.6022	84.8268	2.5859
241.7375	2.5487	200.7488	2.6035	83.8250	2.5271
240.7368	2.5487	199.7419	2.6046	82.8443	2.4592
239.7331	2.5504	198.7464	2.6052	81.8371	2.3884
238.7410	2.5523	197.7565	2.6080	80.8451	2.3192
237.7347	2.5521	196.7453	2.6093	79.8426	2.2619
236.7855	2.5538	195.7562	2.6104	78.8541	2.2156
235.7375	2.5531	194.7569	2.6130	77.8724	2.1843
234.7395	2.5573	193.7507	2.6132	76.8619	2.1601
233.7380	2.5586	192.7498	2.6140	75.8690	2.1425
232.7354	2.5605	191.7543	2.6154	74.8671	2.1295
231.7410	2.5618	190.7461	2.6175	69.9484	2.0818
230.7422	2.5625	189.7446	2.6190	65.0284	2.0443
229.7351	2.5631	184.7777	2.6187	59.9311	2.0199
228.7426	2.5651	179.5733	2.6260	54.8381	2.0023
227.7331	2.5669	174.6131	2.6294	49.7283	1.9849
226.7433	2.5684	169.6093	2.6333	44.6299	1.9668
225.7357	2.5691	164.6014	2.6398	39.5370	1.9479
224.7401	2.5704	159.6257	2.6462	34.4509	1.9281
223.7462	2.5700	154.6287	2.6504	29.3717	1.9054
222.7467	2.5747	149.6279	2.6559	24.2850	1.8733
221.7394	2.5749	144.6423	2.6609	19.2308	1.8176
220.7430	2.5767	139.6324	2.6653	14.1559	1.7206
219.7330	2.5779	134.6558	2.6741	9.1066	1.5289
				3.9993	1.0971

Table S3. Magnetic data for polycrystalline sample of complex **1** in heating mode, second measurement

Heating mode					
**Temperature (K)**	**$\chi_M T$ (cm^3 K mol^{-1})**	**Temperature (K)**	**$\chi_M T$ (cm^3 K mol^{-1})**	**Temperature (K)**	**$\chi_M T$ (cm^3 K mol^{-1})**
3.9939	1.1001	135.2670	2.7143	221.2019	2.5894
9.0895	1.5478	140.2650	2.7025	222.2003	2.5873
14.1699	1.7214	145.2732	2.6998	223.2133	2.5854
19.2432	1.8198	150.2813	2.6947	224.1971	2.5843
24.2871	1.8754	155.3491	2.6898	225.1914	2.5827
29.3811	1.9088	160.3171	2.6824	226.1978	2.5810
34.4637	1.9331	165.3395	2.6762	227.2019	2.5794
39.5378	1.9542	170.3489	2.6708	228.1999	2.5765
44.6236	1.9708	175.3523	2.6643	229.1940	2.5763
49.7120	1.9905	180.3700	2.6582	230.1934	2.5743
54.8035	2.0082	185.3904	2.6521	231.1940	2.5737
59.9038	2.0258	190.3855	2.6443	232.1929	2.5715
65.0012	2.0489	191.2091	2.6309	233.2056	2.5704
70.0881	2.0773	192.2014	2.6297	234.1995	2.5693
75.1051	2.1056	193.1963	2.6274	235.1940	2.5694
76.0811	2.1094	194.1947	2.6270	236.1947	2.5700
77.0840	2.1150	195.1955	2.6238	237.2001	2.5640
78.0874	2.1203	196.1919	2.6233	238.1829	2.5622
79.0858	2.1261	197.2000	2.6210	239.1918	2.5612
80.0910	2.1328	198.1977	2.6217	240.1966	2.5585
81.0850	2.1387	199.1971	2.6196	241.2000	2.5580
82.0792	2.1475	200.1983	2.6184	242.1929	2.5566
83.0888	2.1556	201.2011	2.6156	243.1990	2.5553
84.0870	2.1657	202.2043	2.6147	244.1973	2.5534
85.0841	2.1811	203.1996	2.6154	245.1980	2.5522
86.0934	2.2021	204.2011	2.6127	246.1999	2.5510
87.1051	2.2373	205.1973	2.6117	247.1933	2.5508
88.1076	2.2863	206.1932	2.6093	248.2045	2.5491
89.1136	2.3697	207.2091	2.6075	249.1914	2.5494
90.1165	2.4943	208.1948	2.6075	250.1992	2.5489
91.0909	2.6220	209.1905	2.6066	250.0069	2.5444
92.1092	2.6921	210.2031	2.6043	255.4198	2.5467
93.1095	2.7170	211.2062	2.6029	260.4338	2.5402
94.1031	2.7253	212.2073	2.6026	265.4481	2.5329
95.1048	2.7303	213.2060	2.6009	270.4337	2.5262
100.1849	2.7426	214.1964	2.5999	275.4362	2.5198
105.1894	2.7437	215.1991	2.5991	280.4412	2.5106
110.1854	2.7402	216.1966	2.5964	285.4367	2.5014
115.2340	2.7386	217.1922	2.5951	290.4439	2.4951
120.2296	2.7333	218.2001	2.5926	295.4395	2.4903
125.2524	2.7292	219.1974	2.5918	300.4723	2.4852
130.2137	2.7196	220.1997	2.5913		

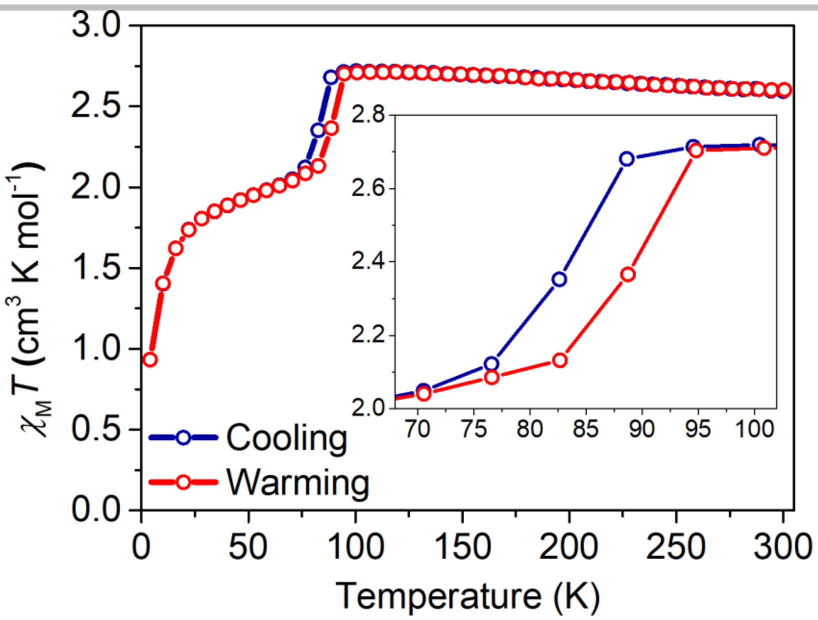

Figure S1: Plot of $\chi_M T$ versus temperature, T for complex **1** in cooling (blue curve) and heating (red curve) modes between 300 K and 4 K measured at 5 K intervals with the inset showing the 8 K wide hysteretic transition.

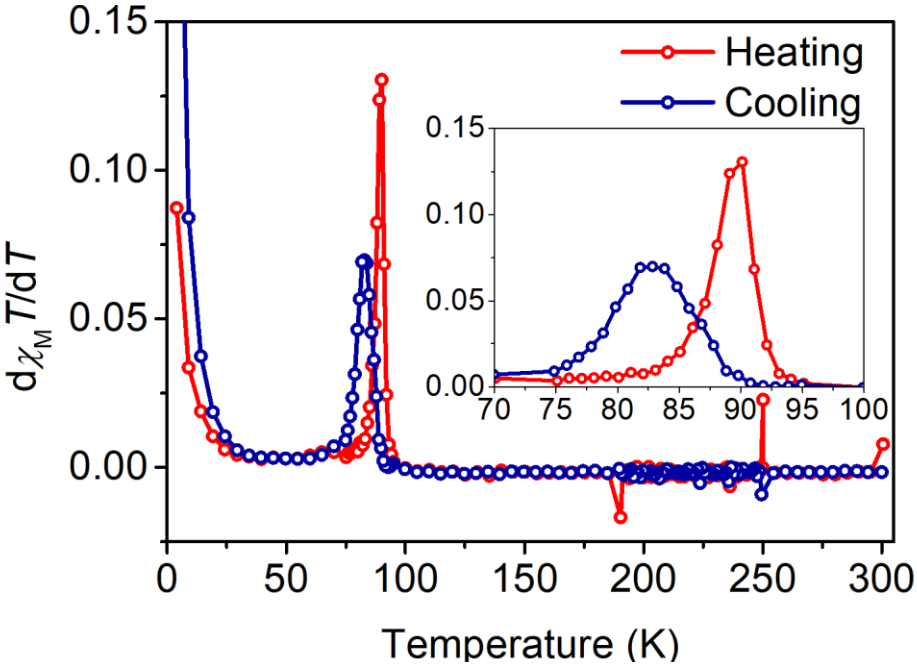

Figure S2: Derivative of $\chi_M T$ versus temperature, T for complex **1** showing discontinuities with $d\chi_M T/T$ maximum values at 82 K (cooling) and 90 K (heating) respectively.

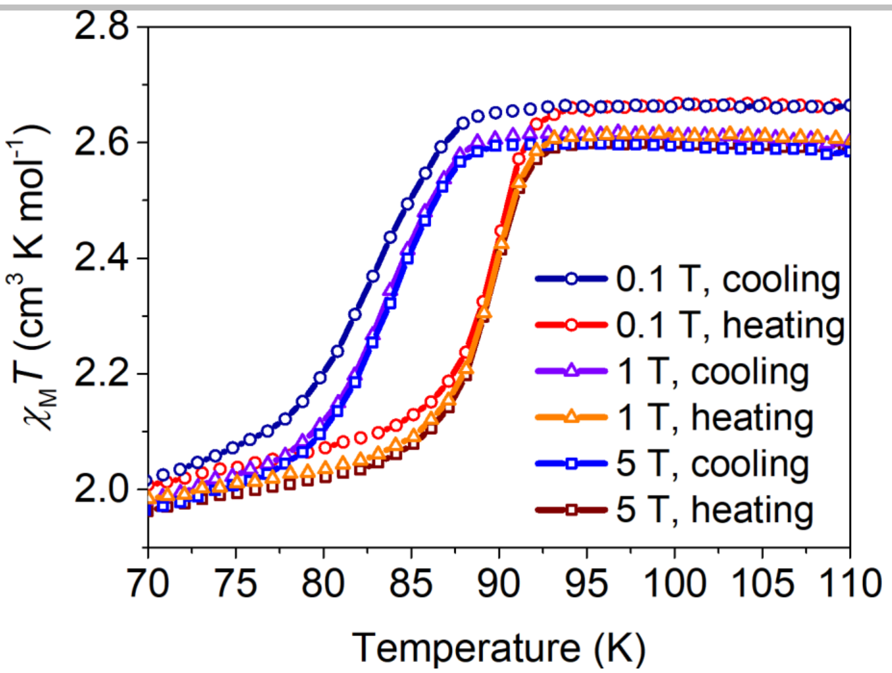

Figure S3: Influence of magnetic field on hysteresis window between 70 K and 110 K measured at 0.1 T, 1 T and 5 T.

3. Single crystal X-ray diffraction

3.1 Crystal and structure refinement data

Table S4. Crystallographic data for complex **1** at 25 K, 83 K, 110 K, 150 K, 250 K and 293 K

	**1,** 25 K	**1,** 83 K	**1,** 110 K	**1,** 150 K	**1,** 250 K	**1,** 293 K
Identification code	MndiBr025K	MndiBr083K	MndiBr110K	MndiBr150K	MndiBr250K	MndiBr293K
Empirical formula	$C_{46}H_{44}BBr_4MnN_4O_2$	$C_{46}H_{44}BBr_4MnN_4O_2$	$C_{46}H_{44}BBr_4MnN_4O_2$	$C_{46}H_{44}BBr_4MnN_4O_2$	$C_{46}H_{44}BBr_4MnN_4O_2$	$C_{46}H_{44}BBr_4MnN_4O_2$
Molecular formula	$[C_{22}H_{24}N_4O_2MnBr_4]^+$ $[C_{24}H_{20}B]^-$	$[C_{22}H_{24}N_4O_2MnBr_4]^+$ $[C_{24}H_{20}B]^-$	$[C_{22}H_{24}N_4O_2MnBr_4]^+$ $[C_{24}H_{20}B]^-$	$[C_{22}H_{24}N_4O_2MnBr_4]^+$ $[C_{24}H_{20}B]^-$	$[C_{22}H_{24}N_4O_2MnBr_4]^+$ $[C_{24}H_{20}B]^-$	$[C_{22}H_{24}N_4O_2MnBr_4]^+$ $[C_{24}H_{20}B]^-$
Formula weight	1070.24	1070.24	1070.24	1070.24	1070.24	1070.24
Wavelength (Å)	1.54184	1.54184	1.54184	1.54184	1.54184	0.71073
Crystal system	Triclinic	Triclinic	Monoclinic	Monoclinic	Monoclinic	Monoclinic
Space group	P1	P1	Pc	Pc	Cc	Cc
a (Å)	14.1317(6)	14.1644(3)	14.86749(12)	14.87250(10)	14.9312(3)	14.9671(4)
b (Å)	23.2539(7)	23.2476(4)	22.71089(12)	22.75550(10)	22.8858(4)	22.9481(5)
c (Å)	13.4869(4)	13.5125(2)	14.14859(13)	14.1802(2)	14.2892(4)	14.3590(4)
α (°)	86.387(2)	86.3840(10)	90	90	90	90
β (°)	103.852(3)	103.918(2)	116.4274(11)	116.4640(10)	116.636(3)	116.674(4)
γ (°)	99.921(3)	100.058(2)	90	90	90	90
Volume ($Å^3$)	4237.7(3)	4251.47(14)	4278.09(7)	4296.15(8)	4364.6(2)	4407.0(2)
Z	4	4	4	4	4	4
Density (calculated) (mg/m^3)	1.677	1.672	1.662	1.655	1.629	1.613
Absorption coefficient (mm^{-1})	7.297	7.273	7.228	7.198	7.085	3.969
Crystal size (mm^3)	0.121 × 0.117 × 0.058	0.152 × 0.113 × 0.062	0.136 × 0.121 × 0.093	0.136 × 0.121 × 0.093	0.136 × 0.121 × 0.093	0.467 × 0.395 × 0.205
Goodness–of–fit on F^2	1.013	1.01	1.02	1.024	1.038	1.054
Final R indices [I>2sigma(I)]	R_1 = 0.0932, wR_2 = 0.2440	R_1 = 0.0448, wR_2 = 0.1149	R_1 = 0.0228, wR_2 = 0.0512	R_1 = 0.0268, wR_2 = 0.0619	R_1 = 0.0320, wR_2 = 0.0794	R_1 = 0.0360, wR_2 = 0.0920
Absolute structure parameter	-0.025(7)	0.001(4)	0.005(2)	0.009(2)	0.000(3)	0.013(9)

Table S5. Selected bond lengths (Å) and angles (°) for complex **1**

	**1** (25 K)	**1** (83 K)	**1** (110 K)	**1** (150 K)	**1** (250 K)	**1** (293 K)
Mn1-O1$_{phen}$	1.868(14)	1.875(7)	1.868(2)	1.866(3)	1.864(3)	1.866(3)
Mn1-O2$_{phen}$	1.908(15)	1.892(7)	1.879(2)	1.879(3)	1.879(3)	1.876(4)
Mn1-N1$_{imine}$	2.008(18)	2.002(8)	2.101(3)	2.098(3)	2.102(4)	2.104(4)
Mn1-N2$_{amine}$	2.08(2)	2.082(9)	2.262(3)	2.259(4)	2.232(5)	2.230(5)
Mn1-N3$_{amine}$	2.058(16)	2.059(8)	2.209(3)	2.206(4)	2.218(5)	2.221(5)
Mn1-N4$_{imine}$	1.99(2)	2.002(10)	2.094(3)	2.090(4)	2.075(5)	2.079(5)
Mn2-O3$_{phen}$	1.903(14)	1.880(7)	1.874(2)	1.874(3)	-	-
Mn2-O4$_{phen}$	1.880(15)	1.890(7)	1.880(2)	1.879(3)	-	-
Mn2-N5$_{imine}$	2.10(2)	2.126(9)	2.126(3)	2.119(4)	-	-
Mn2-N6$_{amine}$	2.24(2)	2.244(10)	2.224(3)	2.216(4)	-	-
Mn2-N7$_{amine}$	2.26(2)	2.261(9)	2.257(3)	2.252(4)	-	-
Mn2-N8$_{imine}$	2.064(19)	2.081(10)	2.078(3)	2.076(4)	-	-
Mn3-O5$_{phen}$	1.862(15)	1.869(7)	-	-	-	-
Mn3-O6$_{phen}$	1.886(16)	1.890(7)	-	-	-	-
Mn3-N9$_{imine}$	2.117(18)	2.113(8)	-	-	-	-
Mn3-N10$_{amine}$	2.21(2)	2.252(10)	-	-	-	-
Mn3-N11$_{amine}$	2.208(17)	2.223(9)	-	-	-	-
Mn3-N12$_{imine}$	2.09(2)	2.091(10)	-	-	-	-
Mn4-O7$_{phen}$	1.904(15)	1.888(7)	-	-	-	-
Mn4-O8$_{phen}$	1.883(15)	1.884(7)	-	-	-	-
Mn4-N13$_{imine}$	2.025(18)	2.034(8)	-	-	-	-
Mn4-N14$_{amine}$	2.07(2)	2.127(10)	-	-	-	-
Mn4-N15$_{amine}$	2.080(19)	2.117(9)	-	-	-	-
Mn4-N16$_{imine}$	1.98(2)	2.018(10)	-	-	-	-
O1-Mn1-O2	176.2(7)	176.3(3)	175.44(12)	175.71(13)	176.59(17)	176.52(18)
O1-Mn1-N1	89.9(8)	89.9(3)	88.74(11)	88.62(13)	87.99(15)	88.01(16)
O1-Mn1-N2	92.3(8)	92.2(3)	94.98(12)	94.81(13)	94.09(17)	93.96(19)
O1-Mn1-N3	85.5(7)	85.6(3)	85.73(11)	85.74(13)	85.84(17)	85.96(18)
O1-Mn1-N4	89.8(8)	89.9(3)	89.42(12)	89.50(13)	89.95(16)	90.31(17)
O2-Mn1-N1	93.8(8)	93.8(3)	92.91(11)	93.00(13)	92.91(16)	92.78(17)
O2-Mn1-N2	88.8(7)	88.8(3)	89.47(12)	89.36(13)	89.29(17)	89.51(18)
O2-Mn1-N3	91.0(7)	90.9(3)	94.14(11)	94.05(13)	94.40(17)	94.41(18)
O2-Mn1-N4	88.7(7)	88.7(3)	86.02(11)	86.21(13)	86.68(16)	86.26(16)
N1-Mn1-N2	85.7(8)	86.2(4)	81.66(12)	81.77(14)	81.8(2)	81.8(2)
N1-Mn1-N3	169.3(8)	169.6(4)	159.49(13)	159.57(14)	159.6(2)	159.8(2)
N2-Mn1-N3	84.8(7)	84.6(4)	79.16(12)	79.16(14)	79.3(2)	79.4(2)
N4-Mn1-N1	100.4(8)	100.0(4)	112.77(12)	112.75(14)	112.83(18)	112.67(19)
N4-Mn1-N2	173.5(7)	173.5(3)	165.05(11)	165.00(13)	165.0(2)	165.0(2)

N4-Mn1-N3	89.3(7)	89.4(4)	86.94(12)	86.85(14)	86.6(2)	86.6(2)
O3-Mn2-O4	176.4(7)	176.2(3)	176.77(12)	176.78(14)	-	-
O3-Mn2-N5	88.1(7)	87.6(3)	87.67(11)	87.73(13)	-	-
O3-Mn2-N6	95.1(7)	95.3(3)	94.42(12)	94.27(13)	-	-
O3-Mn2-N7	84.4(7)	85.1(3)	85.18(11)	85.21(13)	-	-
O3-Mn2-N8	90.4(7)	90.0(3)	89.90(12)	89.95(13)	-	-
O4-Mn2-N5	92.0(7)	92.4(3)	92.47(11)	92.50(13)	-	-
O4-Mn2-N6	88.5(7)	88.5(3)	88.79(12)	88.93(13)	-	-
O4-Mn2-N7	96.7(7)	96.2(3)	95.82(11)	95.69(13)	-	-
O4-Mn2-N8	86.3(7)	86.5(3)	87.11(12)	87.04(13)	-	-
N5-Mn2-N6	82.8(8)	81.4(4)	81.52(13)	81.53(15)	-	-
N5-Mn2-N7	158.6(8)	157.6(4)	158.11(13)	158.26(15)	-	-
N7-Mn2-N6	77.9(8)	78.2(4)	78.44(14)	78.54(16)	-	-
N8-Mn2-N5	115.2(7)	116.1(3)	114.97(13)	114.75(14)	-	-
N8-Mn2-N6	161.4(7)	161.9(4)	163.15(13)	163.37(15)	-	-
N8-Mn2-N7	84.9(7)	85.1(4)	85.72(13)	85.82(15)	-	-
O5-Mn3-O6	175.4(8)	175.3(4)	-	-	-	-
O5-Mn3-N9	88.0(7)	88.3(3)	-	-	-	-
O5-Mn3-N10	95.5(7)	94.8(3)	-	-	-	-
O5-Mn3-N11	85.7(6)	85.7(3)	-	-	-	-
O5-Mn3-N12	88.8(7)	89.6(3)	-	-	-	-
O6-Mn3-N9	93.2(7)	92.6(3)	-	-	-	-
O6-Mn3-N10	89.1(7)	89.9(3)	-	-	-	-
O6-Mn3-N11	94.7(6)	95.0(3)	-	-	-	-
O6-Mn3-N12	86.7(7)	85.8(3)	-	-	-	-
N9-Mn3-N10	82.2(7)	82.0(3)	-	-	-	-
N9-Mn3-N11	159.0(7)	159.1(4)	-	-	-	-
N11-Mn3-N10	78.5(7)	78.5(3)	-	-	-	-
N12-Mn3-N9	112.3(8)	112.7(3)	-	-	-	-
N12-Mn3-N10	165.0(7)	164.8(3)	-	-	-	-
N12-Mn3-N11	87.5(7)	87.3(3)	-	-	-	-
O7-Mn4-O8	176.2(6)	176.2(3)	-	-	-	-
O7-Mn4-N13	90.1(7)	89.3(3)	-	-	-	-
O7-Mn4-N14	94.7(7)	94.5(3)	-	-	-	-
O7-Mn4-N15	85.6(7)	85.2(3)	-	-	-	-
O7-Mn4-N16	88.8(7)	89.6(4)	-	-	-	-
O8-Mn4-N13	93.5(7)	94.1(3)	-	-	-	-
O8-Mn4-N14	86.8(7)	87.6(3)	-	-	-	-
O8-Mn4-N15	91.1(7)	92.0(3)	-	-	-	-
O8-Mn4-N16	89.3(7)	87.8(3)	-	-	-	-

N13-Mn4-N14	84.9(8)	83.5(4)	-	-	-	-
N13-Mn4-N15	167.6(8)	164.6(4)	-	-	-	-
N15-Mn4-N14	83.9(8)	82.6(4)	-	-	-	-
N16-Mn4-N13	102.2(8)	106.0(4)	-	-	-	-
N16-Mn4-N14	172.1(7)	169.7(4)	-	-	-	-
N16-Mn4-N15	89.3(8)	88.4(4)	-	-	-	-

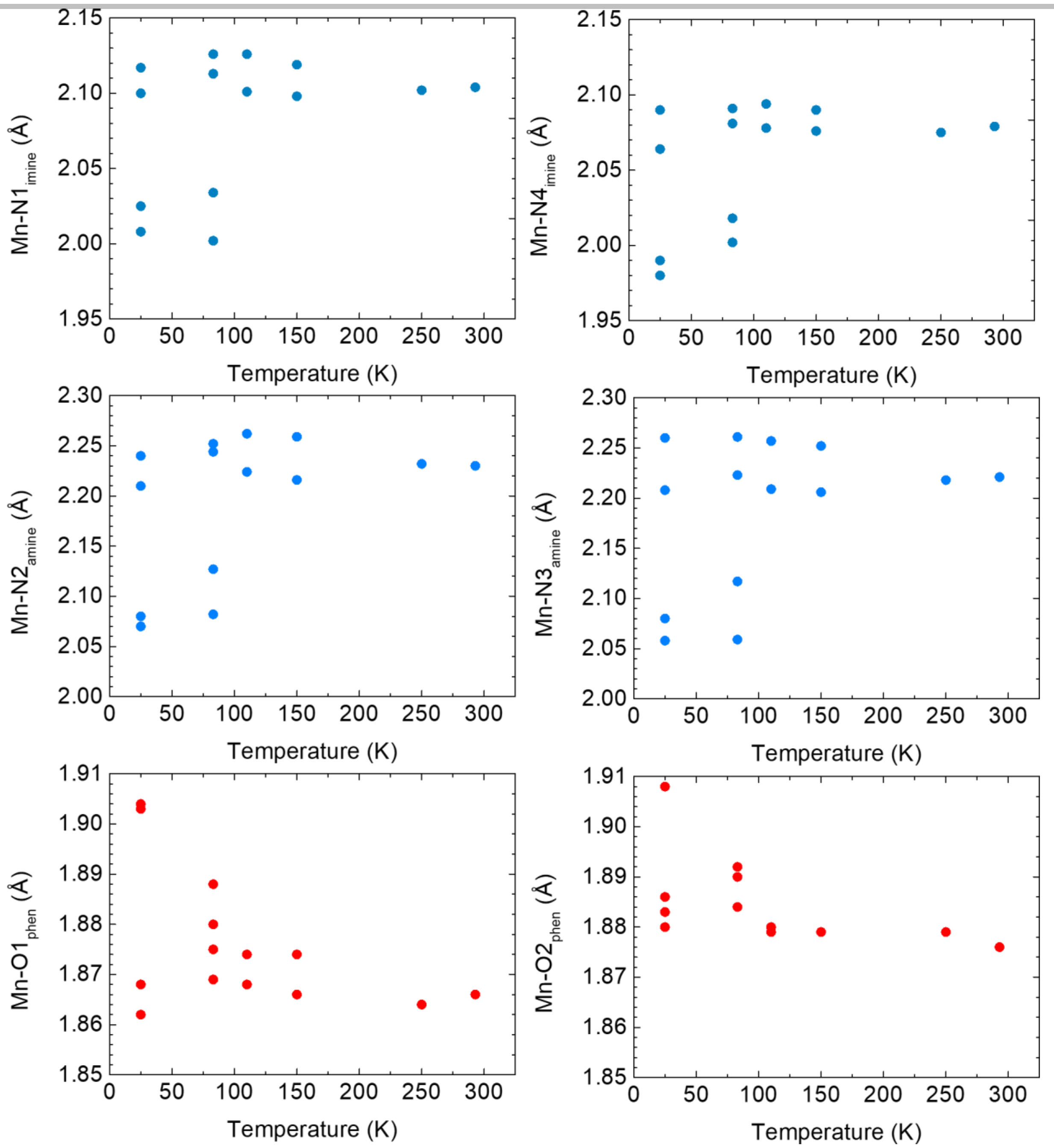

Figure S4: Evolution of bond lengths between the nitrogen and oxygen donor atoms in the first coordination sphere to the Mn^{3+} cation(s) of complex **1** measured at 293 K, 250 K, 150 K, 110 K, 83 K and 25 K.

A small increase in bond lengths of Mn-N_{amine}, Mn-N_{imine} and Mn-O_{phen} is observed going from the HT structures at 293 K and 250 K to the INT structures at 150 K and 110 K. Upon cooling further down, the LT structures at 83 K and 25 K sees an overall increase in Mn-O_{phen} bond lengths compared to the INT structures at 110 K and 150 K. For Mn-$N1_{imine}$, Mn-$N2_{amine}$, and Mn-$N3_{amine}$, the bond lengths remain more or less the same at 83 K and 25 K for both S = 2 Mn^{3+} cations as compared to the bond lengths observed at 110 K. A decrease in bond lengths is observed for the S = 1 Mn^{3+} cations due to the depopulation of the $d_{x^2-y^2}$ orbital of the anti-bonding e_g* orbitals in the S = 1 state.

Table S6. Calculated distortion parameters and list of spin states for each asymmetric [MnL]$^{+}$ cation in complex 1 at 25 K, 83 K, 110 K, 150 K, 250 K and 293 K. Z' is the number of Mn^{3+} sites in the asymmetric unit.

**Temperature**		**25 K**	**83 K**	**110 K**	**150 K**	**250 K**	**293 K**
**Space group**		***P1***	***P1***	***Pc***	***Pc***	***Cc***	***Cc***
**Z'** [a]		**4**	**4**	**2**	**2**	**1**	**1**
Parameter	Mn site						
ΣMn (°)[b]	Mn1	34.88	33.72	67.67	67.41	66.89	66.50
	Mn2	76.42	77.57	73.38	72.47	-	-
	Mn3	69.29	67.99	-	-	-	-
	Mn4	43.13	52.63	-	-	-	-
Φ (°)[c]	Mn1	117.72	113.68	259.89 278.87	257.63 275.10	253.49	251.50
	Mn2	287.88	295.61	-	-	-	-
	Mn3	263.73	264.03	-	-	-	-
	Mn4	143.12	180.41			-	-
ζ (Å)[d]	Mn1	0.388	0.409	0.780 0.785	0.775 0.772	0.759	0.769
	Mn2	0.751	0.783	-	-	-	-
	Mn3	0.749	0.777	-	-	-	-
	Mn4	0.406	0.501			-	-
Spin state	Mn1	S = 1	S = 1	S = 2	S = 2	S = 2	S = 2
	Mn2	S = 2	S = 2	S = 2	S = 2	-	-
	Mn3	S = 2	S = 2	-	-	-	-
	Mn4	S = 1	S = 1	-	-	-	-

[a] Z' is number of independent sites in the asymmetric unit. [b]ΣMn is the sum of the deviation from 90° of the 12 cis-angles of the MnN_4O_2 octahedron. [c]Φ is the sum of the deviation from 60° of the 24 trigonal angles of the projection of the MnN_4O_2 octahedron onto the trigonal faces. [d]ζ is the distance distortion parameter, which is the sum of deviation from individual M-X bond distances with respect to the mean metal-ligand bond distance.[7]

3.2 Crystal structures and packing of complex 1

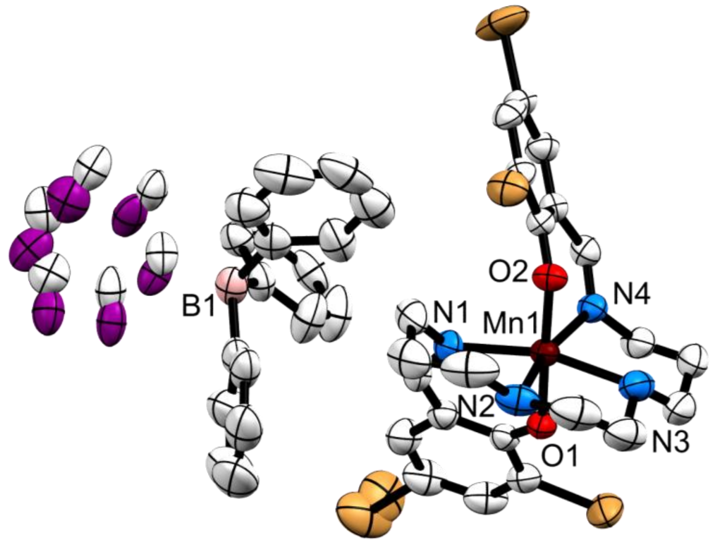

Figure S5: Asymmetric unit of complex **1**, [Mn(3,5-diBr-sal)$_2$323]BPh_4 measured at 293 K shown with 50% probability ellipsoids with hydrogen atoms omitted for clarity. The disordered carbon atoms on the BPh_4^- anion are highlighted in purple.

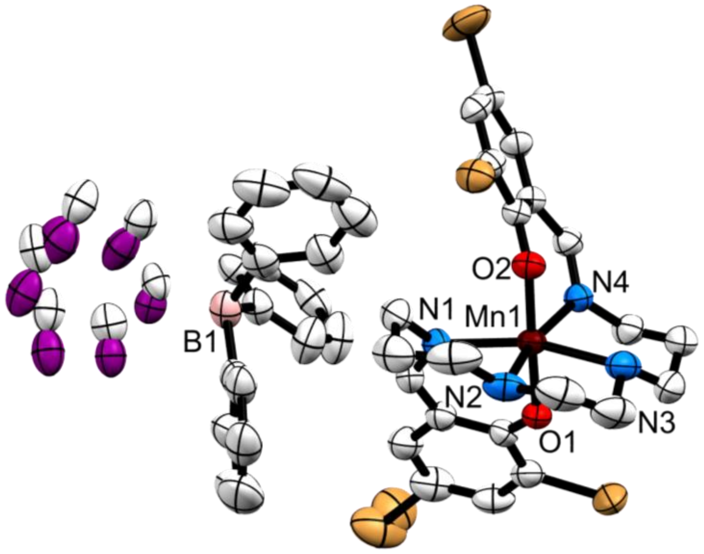

Figure S6: Asymmetric unit of complex **1**, [Mn(3,5-diBr-sal)$_2$323]BPh_4 measured at 250 K shown with 50% probability ellipsoids with hydrogen atoms omitted for clarity. The disordered carbon atoms on the BPh_4^- anion are highlighted in purple.

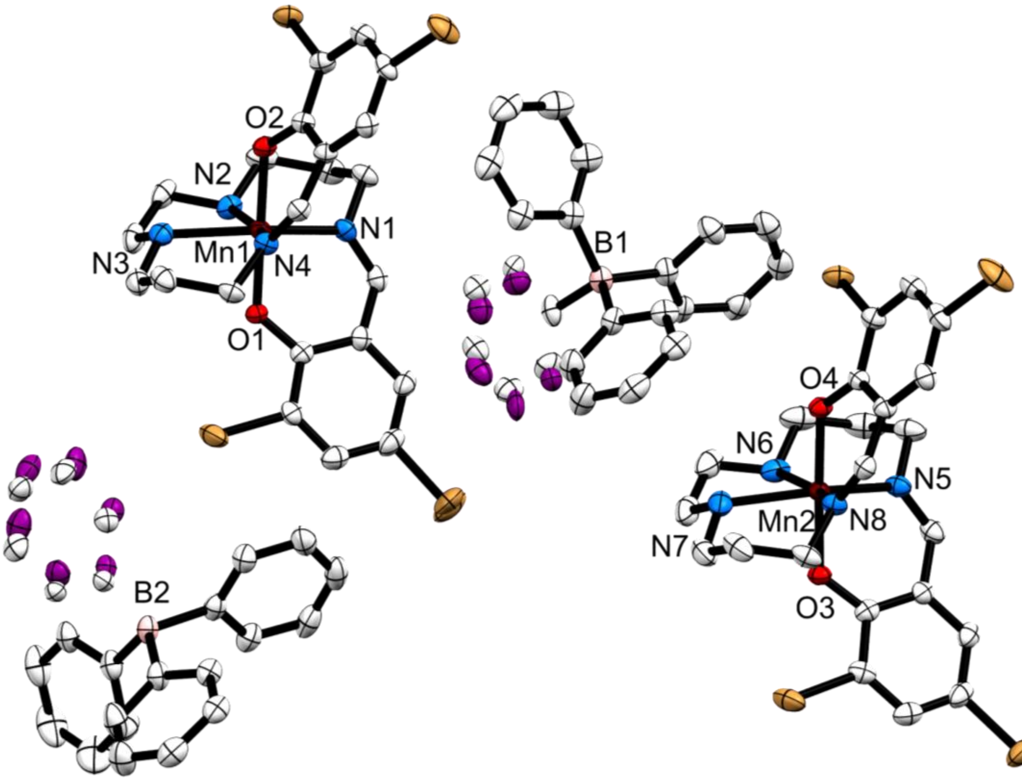

Figure S7: Asymmetric unit of complex **1**, $[Mn(3,5\text{-diBr-sal})_2323]BPh_4$ measured at 150 K shown with 50% probability ellipsoids with hydrogen atoms omitted for clarity. The disordered carbon atoms on the BPh_4^- anion are highlighted in purple.

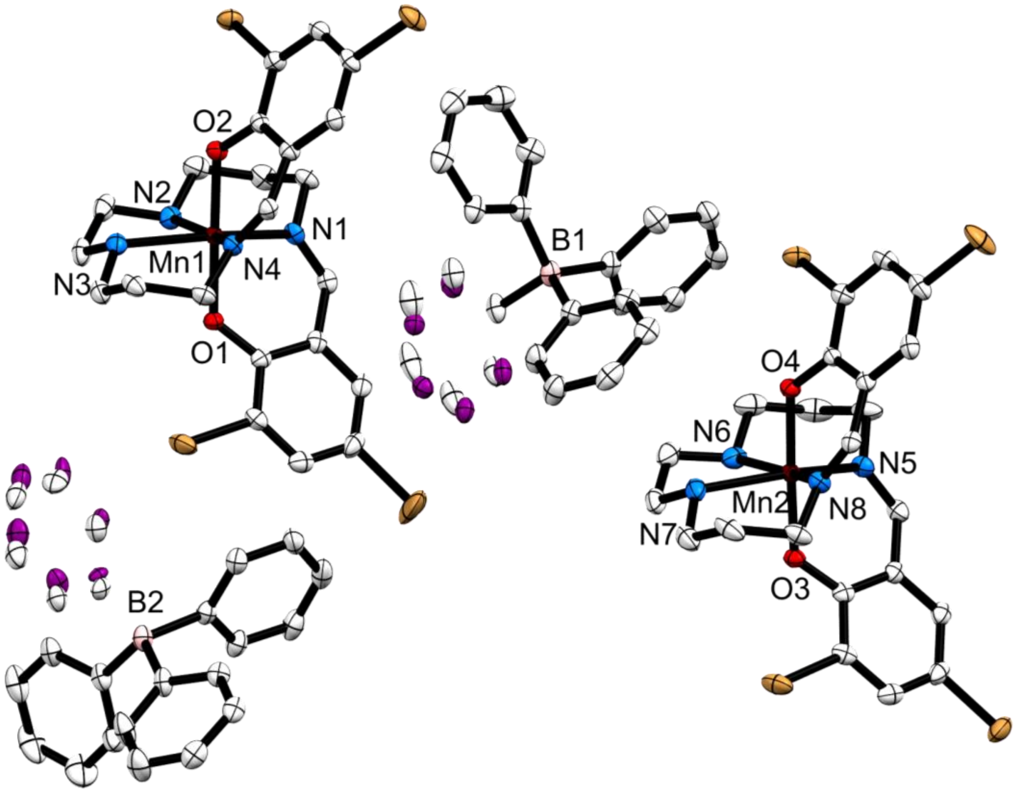

Figure S8: Asymmetric unit of complex **1**, $[Mn(3,5\text{-diBr-sal})_2323]BPh_4$ measured at 110 K shown with 50% probability ellipsoids with hydrogen atoms omitted for clarity. The disordered carbon atoms on the BPh_4^- anion are highlighted in purple.

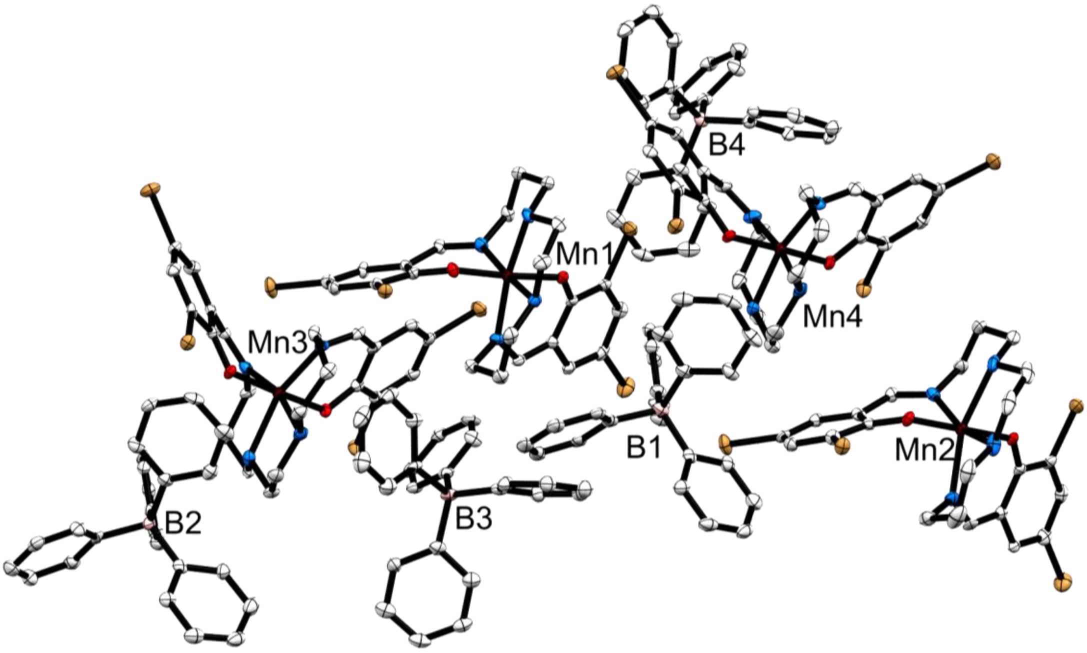

Figure S9: Asymmetric unit of complex **1**, [Mn(3,5-diBr-sal)$_2$323]BPh$_4$ measured at 83 K shown with 50% probability ellipsoids with hydrogen atoms omitted for clarity.

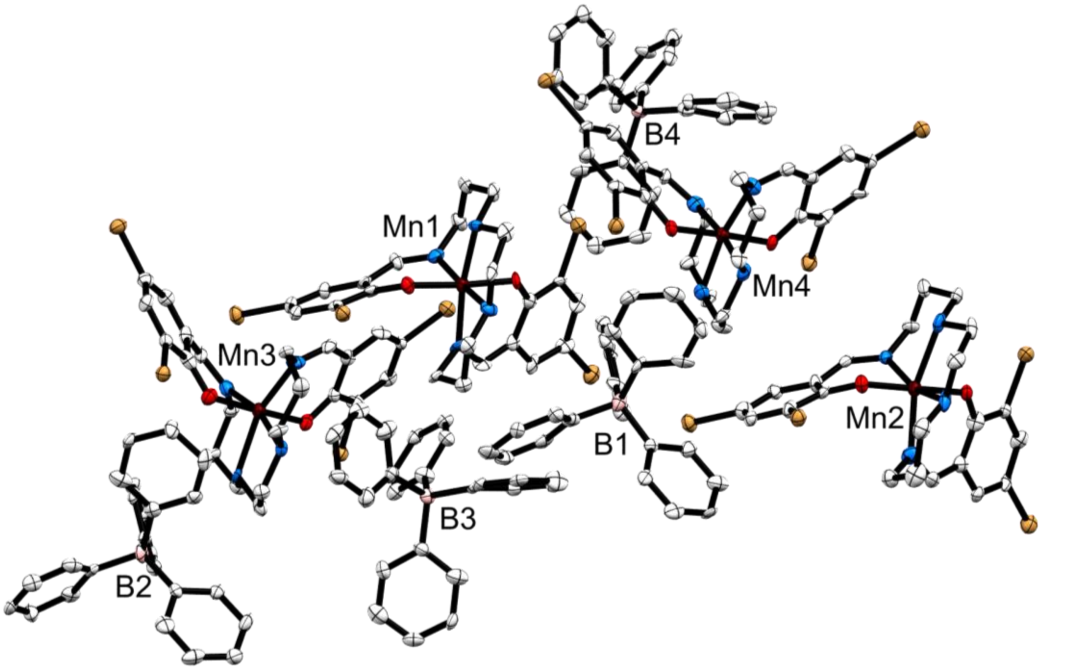

Figure S10: Asymmetric unit of complex **1**, [Mn(3,5-diBr-sal)$_2$323]BPh$_4$ measured at 25 K shown with 50% probability ellipsoids with hydrogen atoms omitted for clarity.

Complex **1** crystallises in the non-centrosymmetric polar space group *Cc* at 293 K, 250 K and 100 K* respectively. At 150 K and 110 K, complex **1** crystallises in the non-centrosymmetric polar space group *Pc* and at 83 K in the non-symmetric chiral and polar space group *P1*. Complex **1** contains the [Mn(3,5-diBr-sal$_2$(323))]$^+$ cation and the tetraphenylborate counteranion, BPh$_4^-$. Each structure contain four Mn^{3+} cations and four BPh$_4^-$ anions in the unit cell. Hence, the *Cc* structures has Z = 4 but Z' = 1, the *Pc* structures has Z = 4 but Z' = 2 and the *P1* structure has Z = 4 and Z' = 4. The structures are all polar and also chiral for the *P1* structure with Flack parameters[8] of 0.013(9) (293 K), 0.000(3) (250 K), 0.009(2) (150 K), 0.006(2) (110 K), and 0.008(7) (83 K). No solvent molecules were located in any of the structures. The tetraphenylborate anions are disordered in the *Cc* structures measured at 293 K, and 250 K and the *Pc* structures at 150 K and 110 K. There is no observed disorder in the BPh$_4^-$ counteranion at 83 K. Figure S11 shows the degree of occupational disorder in the BPh$_4^-$ anions at each given temperature.

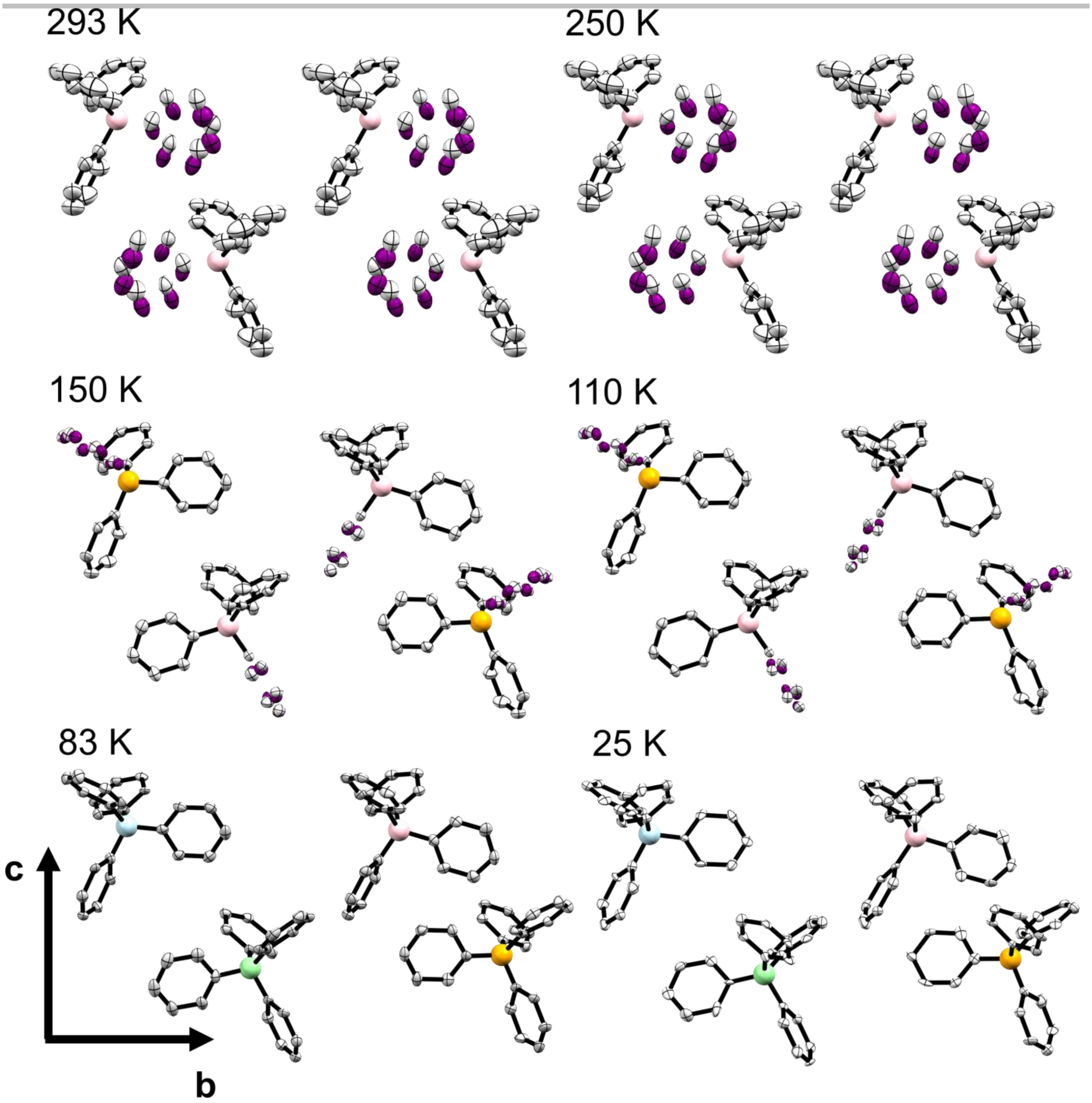

Figure S11: Unit cell content of the tetraphenylborate counteranion BPh_4^- viewed along the *a* axis with respect to the *Cc* cell with the other structures oriented the same way for comparison. The degree of disorder on the phenyl rings across the listed temperature range is highlighted with purple ellipsoids. Structures are shown with 50% probability ellipsoids with the Mn^{3+} chelated cations and hydrogen atoms omitted for clarity.

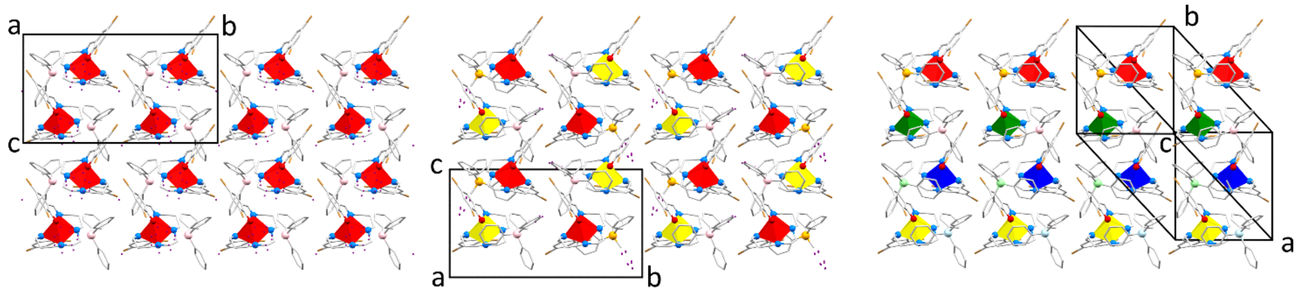

Figure S12: Packing of the unit cell along the a axis for the *Cc* cell of complex **1**, [Mn(3,5-diBr-sal)$_2$323]BPh$_4$ measured at left: 250 K, middle: 110 K, right: 83 K with the Mn^{3+} complex cation shown in a polyhedral environment with $N_4O_2^{2-}$ donor atoms and boron atom in the tetraphenylborate counteranion shown as ball and stick figures.

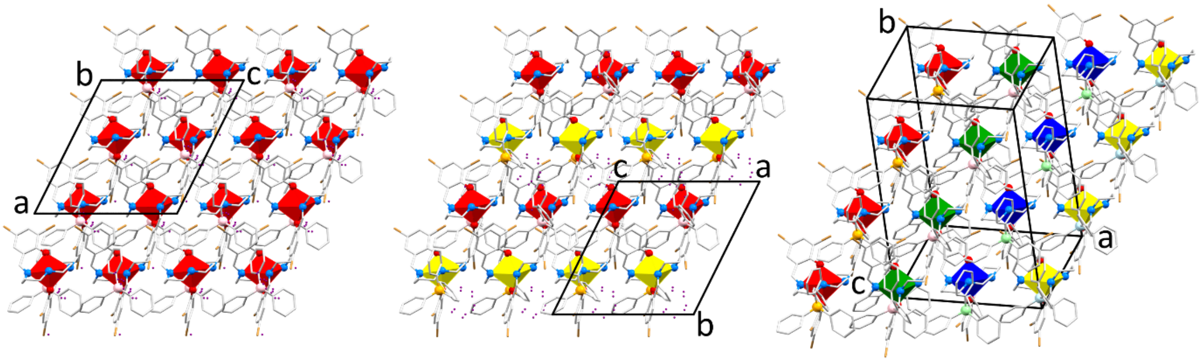

Figure S13: Packing of the unit cell along the b axis for the *Cc* cell of complex **1**, [Mn(3,5-diBr-sal)$_2$323]BPh$_4$ measured at left: 250 K, middle: 110 K, right: 83 K with the Mn^{3+} complex cation shown in a polyhedral environment with $N_4O_2^{2-}$ donor atoms and boron atom in the tetraphenylborate counteranion shown as ball and stick figures.

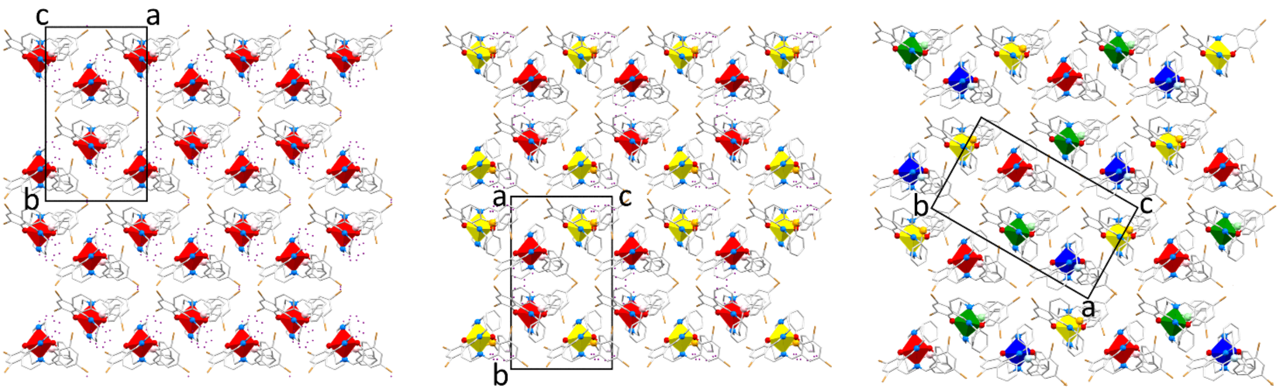

Figure S14: Packing of the unit cell along the c axis for the *Cc* cell of complex **1**, [Mn(3,5-diBr-sal)$_2$323]BPh$_4$ measured at left: 250 K, middle: 110 K, right: 83 K with the Mn^{3+} complex cation shown in a polyhedral environment with $N_4O_2^{2-}$ donor atoms and boron atom in the tetraphenylborate counteranion shown as ball and stick figures.

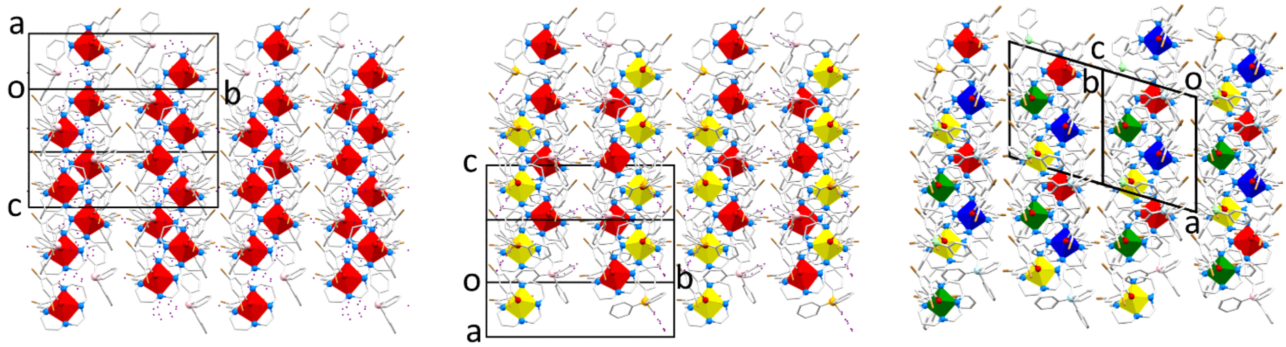

Figure S15: Packing of the unit cell along the a* axis for the *Cc* cell of complex **1**, [Mn(3,5-diBr-sal)$_2$323]BPh$_4$ measured at left: 250 K, middle: 110 K, right: 83 K with the Mn^{3+} complex cation shown in a polyhedral environment with $N_4O_2^{2-}$ donor atoms and boron atom in the tetraphenylborate counteranion shown as ball and stick figures.

In the packing arrangement of each structure measured at 250 K, 110 K and 83 K, the spin state of the $[Mn^{III}L]^+$ complex cation is colour coded by red and yellow (S = 2) and blue and green (S = 1), Figure S12-S15. The spin crossover behaviour is concluded to be associated with the symmetry breaking event going from *Pc* → *P1* and as the monoclinic axis changes direction, this phase transition is reconstructive, without a group-subgroup relationship between the two phases as seen by the striped orders of $[Mn^{III}L]^+$ complex cations in Figure S12-S15. The phase transition is therefore first order and is reversible, since upon heating, the high temperature phase is recovered above 90 K. The presence of hysteresis in the $\chi_M T$ versus *T* is directly related to the reconstructive nature of the phase transition associated with the large structural reorganization in complex **1**.

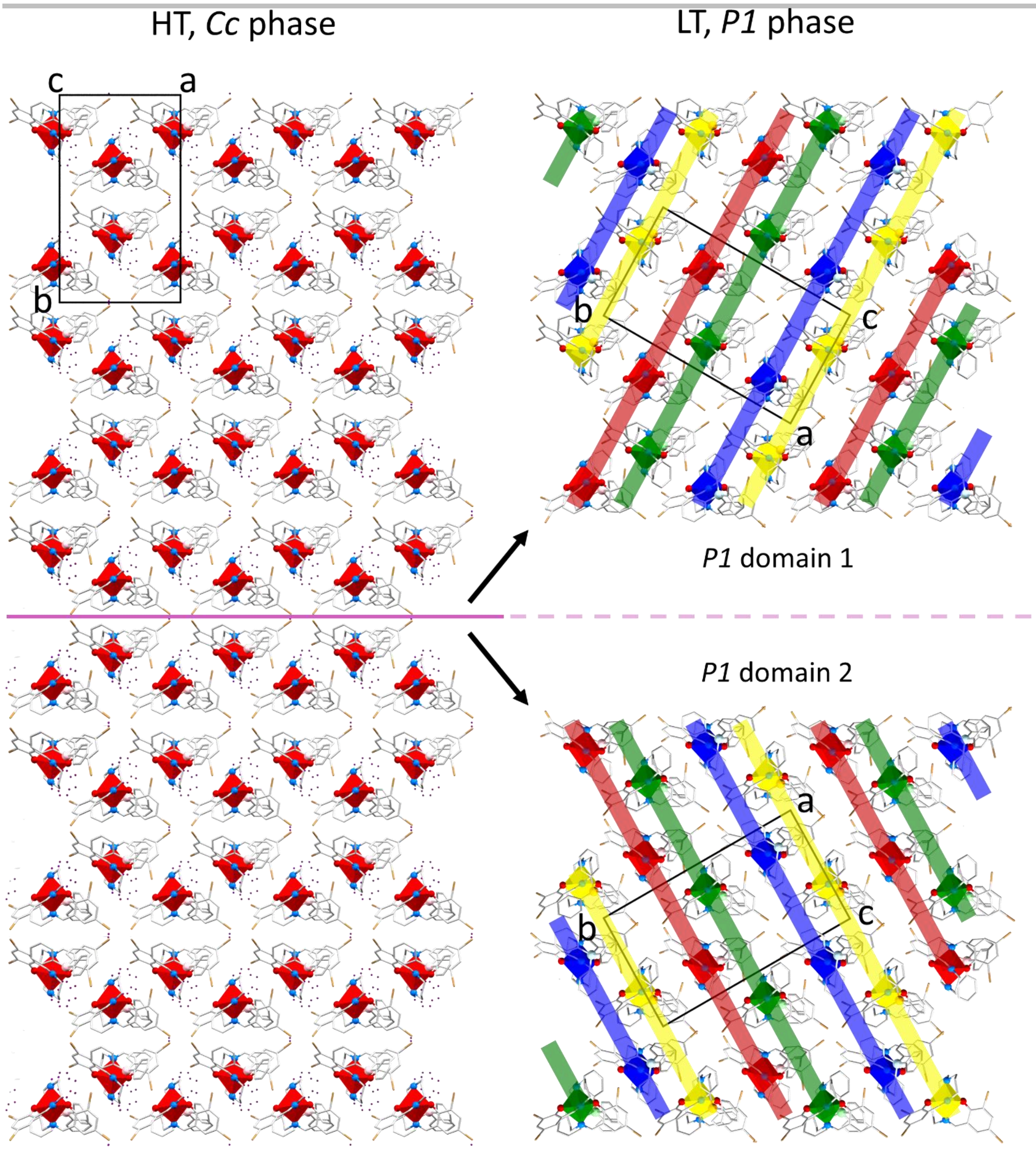

Figure S16: Due to the loss of the monoclinic axis, no more perpendicular to (*a*,*c*), two ferroelastic domains can form in the *P1* phase. The relative molecular packing between the domains are related by the symmetry plane.

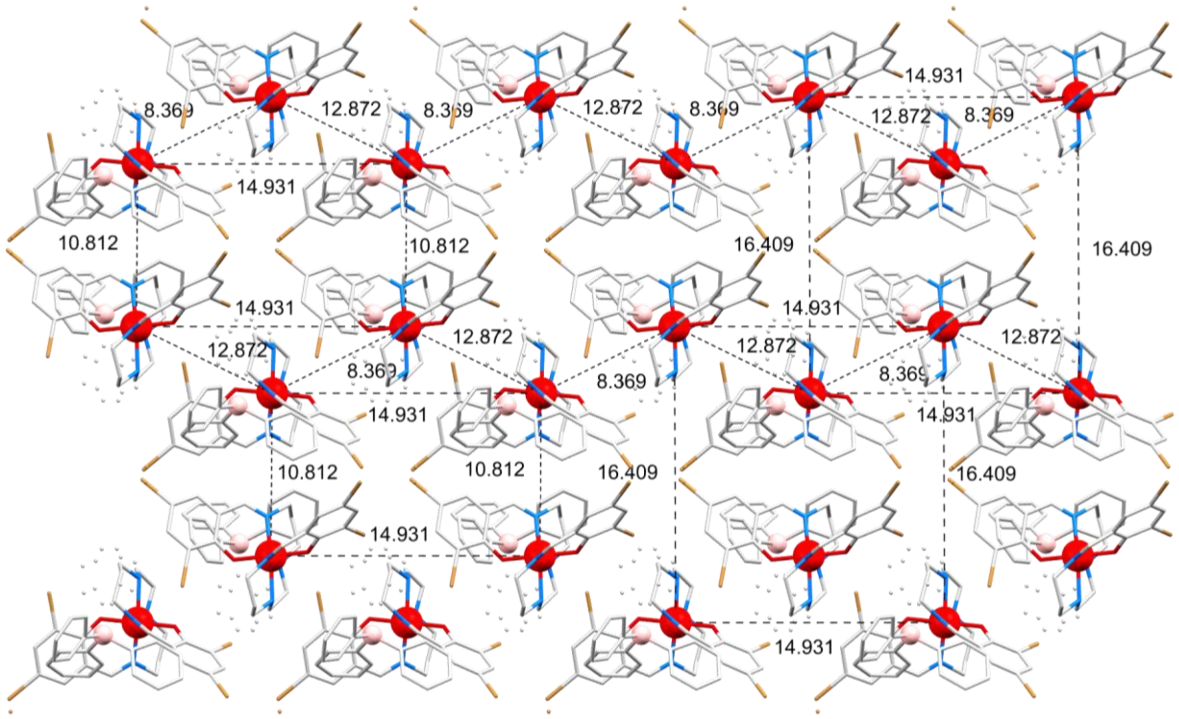

Figure S17: Measured distance between Mn^{3+} complex cations at 250 K viewed along the *c* axis for the *Cc* cell of complex **1**, [Mn(3,5-diBr-sal)$_2$323]BPh_4 represented by capped sticks except Mn^{3+} atoms and boron atoms in the tetraphenylborate anions shown as ball and stick figures.

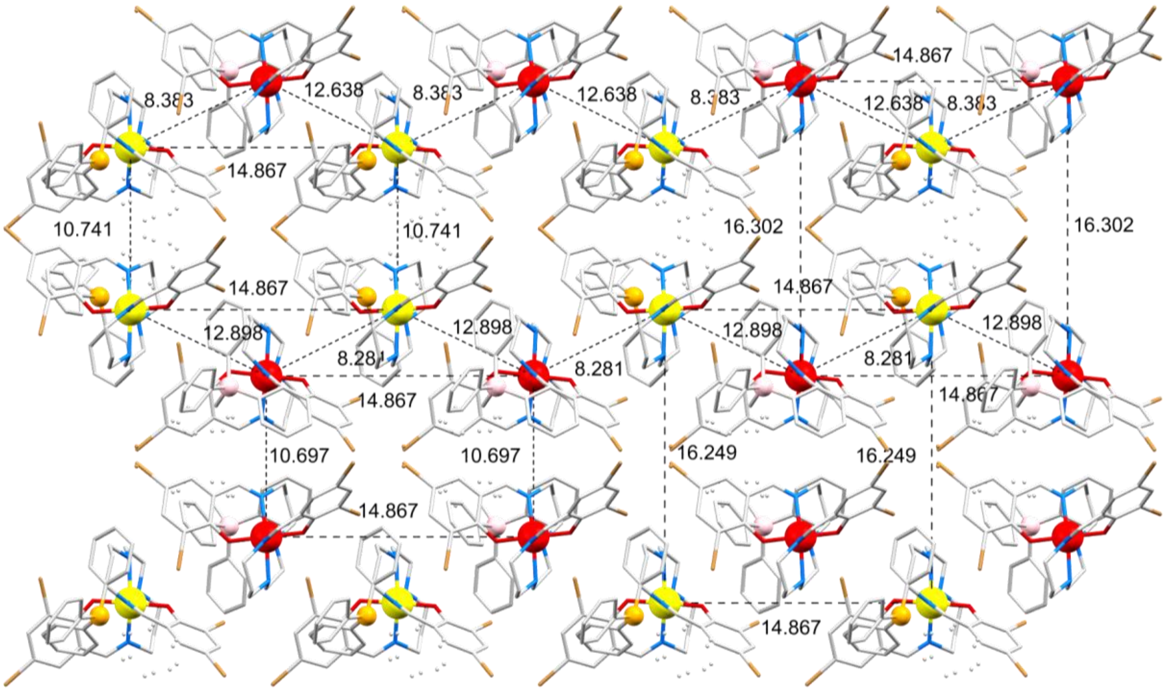

Figure S18: Measured distance between Mn^{3+} complex cations at 110 K viewed along the *c* axis for the *Cc* cell of complex **1**, [Mn(3,5-diBr-sal)$_2$323]BPh_4 represented by capped sticks except Mn^{3+} atoms and boron atoms in the tetraphenylborate anions shown as ball and stick figures.

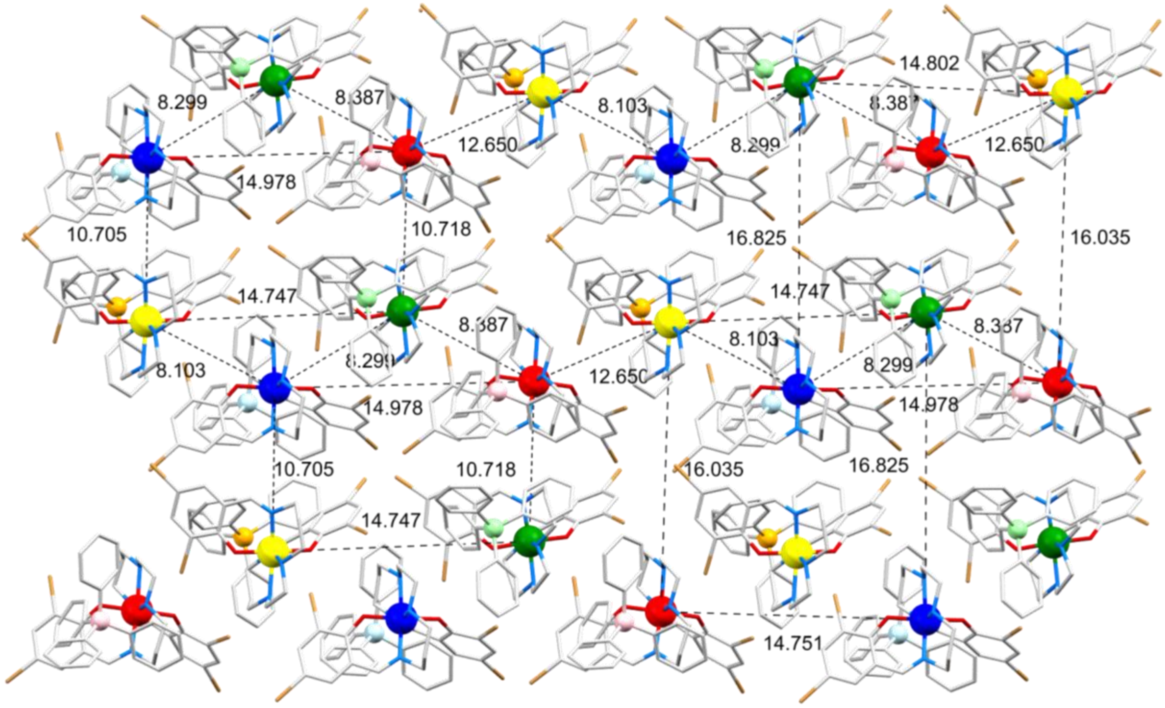

Figure S19: Measured distance between Mn^{3+} complex cations at 83 K viewed along the *c* axis for the *Cc* cell of complex **1**, [Mn(3,5-diBr-sal)$_2$323]BPh_4 represented by capped sticks except Mn^{3+} atoms and boron atoms in the tetraphenylborate anions shown as ball and stick figures.

The measured distances between the Mn^{3+} complex cations in the HT (250 K) and INT (110 K) structures do not differ significantly from each other. However, for the LT (83 K) structure, the distance between the two S = 1 LS Mn^{3+} complex cations (green and blue) change slightly as opposed to the HT (250 K) and INT (110 K) when comparing the intermolecular distances between the Mn^{3+} cations in the structures in the chain running along the *c* axis of the HT structure.

3.3 Specific intermolecular interactions of complex 1

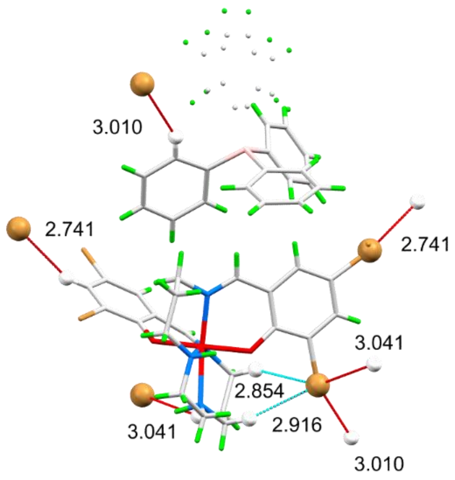

Figure S20: View of weak interactions between the asymmetric unit of [MnL]$^{+}$ cation and tetraphenylborate anion of complex **1** at 250 K shown with capped sticks except atoms involved in weak hydrogen bonding which are represented with ball and stick figures. Hydrogen atoms not involved in hydrogen bonding are highlighted in green.

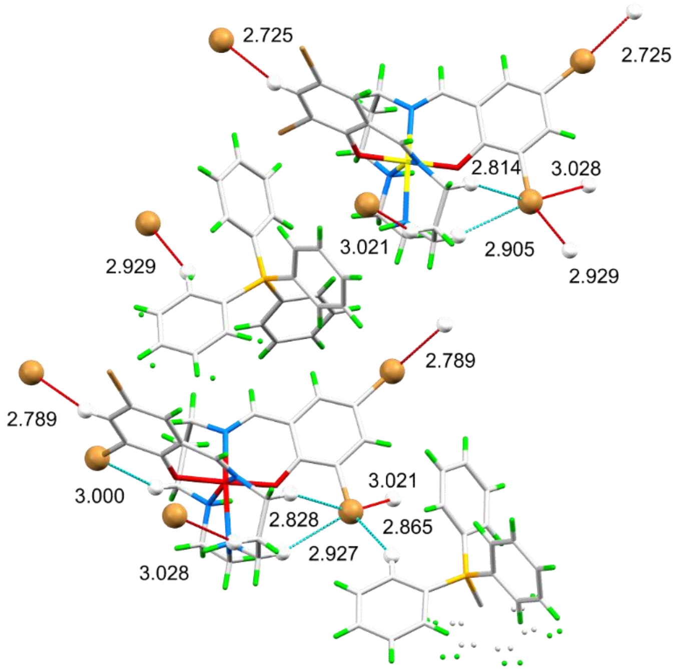

Figure S21: View of weak interactions between the asymmetric unit of [MnL]$^{+}$ cations and tetraphenylborate anions of complex **1** at 110 K shown with capped sticks except atoms involved in weak hydrogen bonding which are represented with ball and stick figures. Hydrogen atoms not involved in hydrogen bonding are highlighted in green.

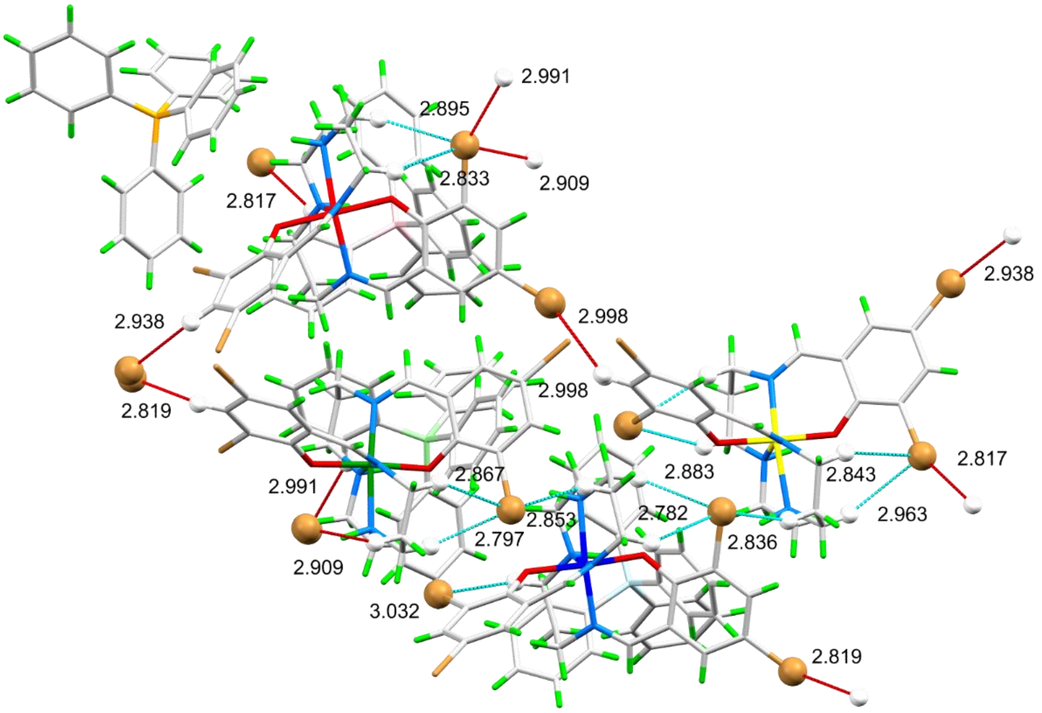

Figure S22: View of weak interactions between the asymmetric unit of $[MnL]^{+}$ cations and tetraphenylborate anions of complex **1** at 83 K shown with capped sticks except atoms involved in weak hydrogen bonding which are represented with ball and stick figures. Hydrogen atoms not involved in hydrogen bonding are highlighted in green.

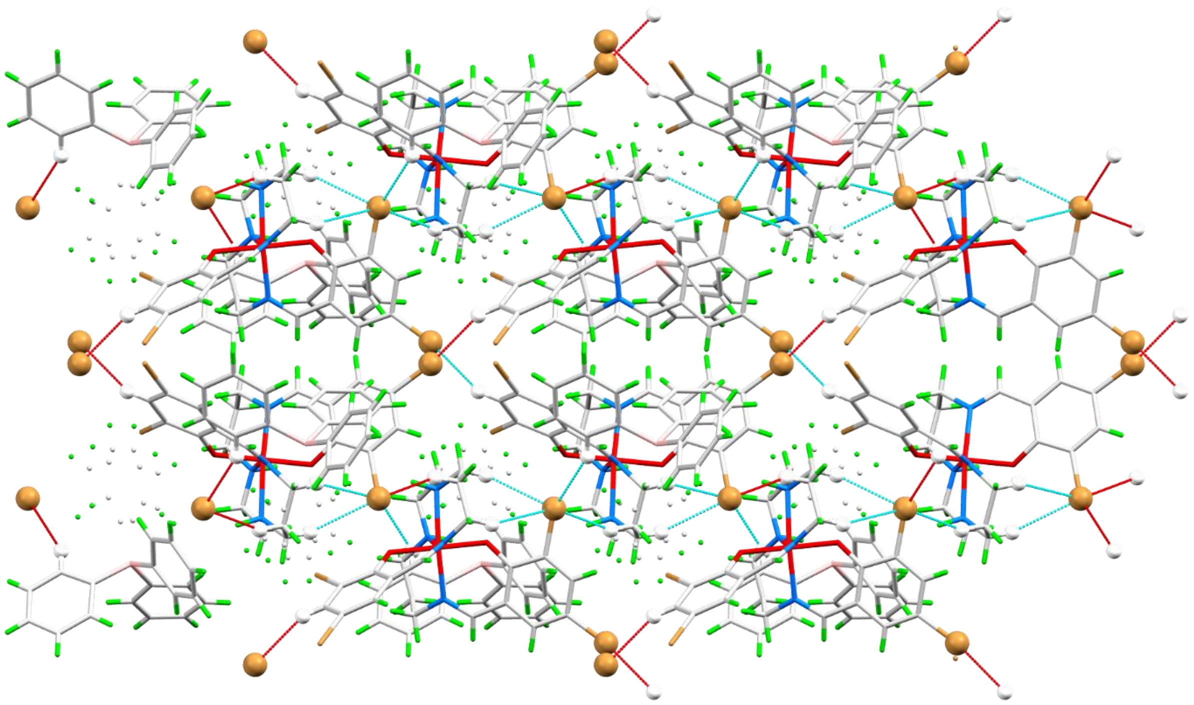

Figure S23: View of packing arrangement with weak interactions between $[MnL]^{+}$ cations and tetraphenylborate anions of complex **1** highlighted with blue dashed lines at 250 K. Atoms not involved in hydrogen bonding are shown with capped sticks and atoms involved in weak hydrogen bonding are represented with ball and stick figures. Hydrogen atoms not involved in hydrogen bonding are highlighted in green.

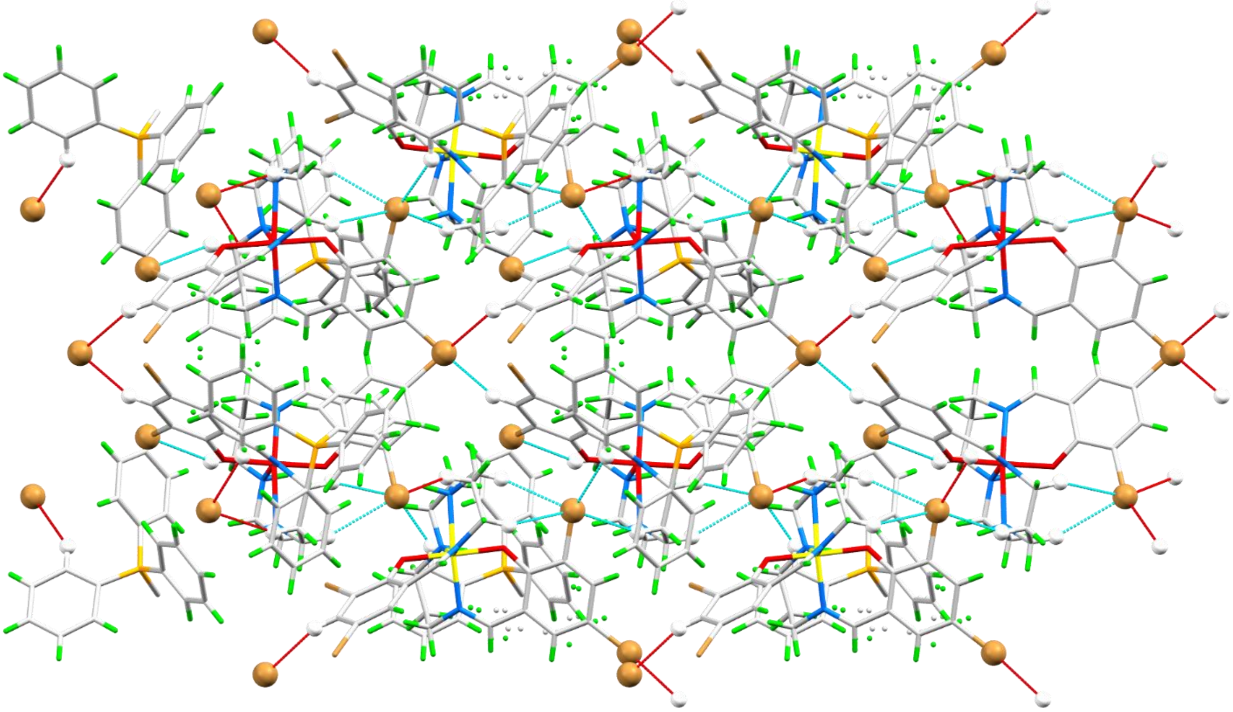

Figure S24: View of packing arrangement with weak interactions between [MnL]$^+$ cations and tetraphenylborate anions of complex **1** highlighted with blue dashed lines at 110 K. Atoms not involved in hydrogen bonding are shown with capped sticks and atoms involved in weak hydrogen bonding are represented with ball and stick figures. Hydrogen atoms not involved in hydrogen bonding are highlighted in green.

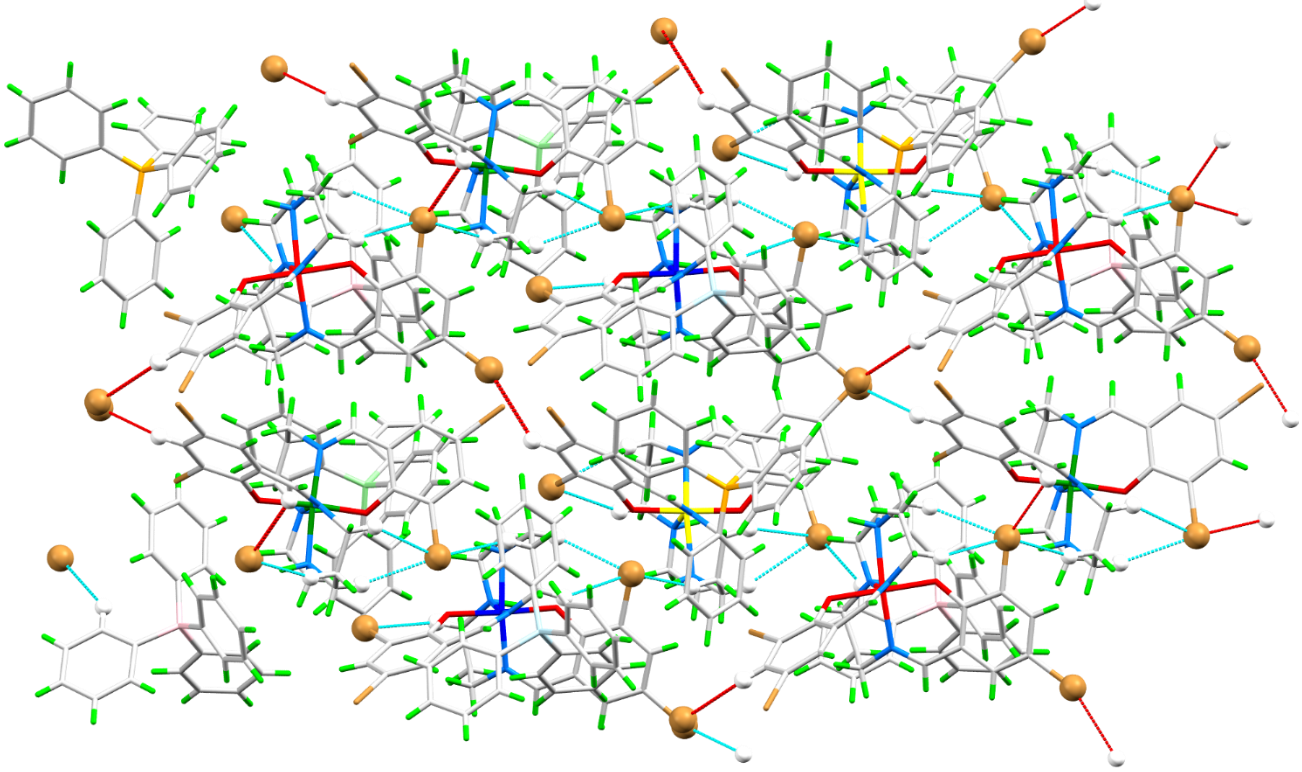

Figure S25: View of packing arrangement with weak interactions between [MnL]$^+$ cations and tetraphenylborate anions of complex **1** highlighted with blue dashed lines at 83 K. Atoms not involved in hydrogen bonding are shown with capped sticks and atoms involved in weak hydrogen bonding are represented with ball and stick figures. Hydrogen atoms not involved in hydrogen bonding are highlighted in green.

3.4 Hirshfeld surface mapping

The Hirshfeld surfaces are mapped with d_{norm}, and 2D fingerprint plots were generated using CrystalExplorer 17.5.[9] The graphical plot uses a red-white-blue colour scheme for the molecular Hirshfeld surfaces. The red highlights on the Hirshfeld surfaces show contacts shorter than the van der Waals distance, contacts within the van der Waals distance is shown in white and longer contacts are shown in blue.

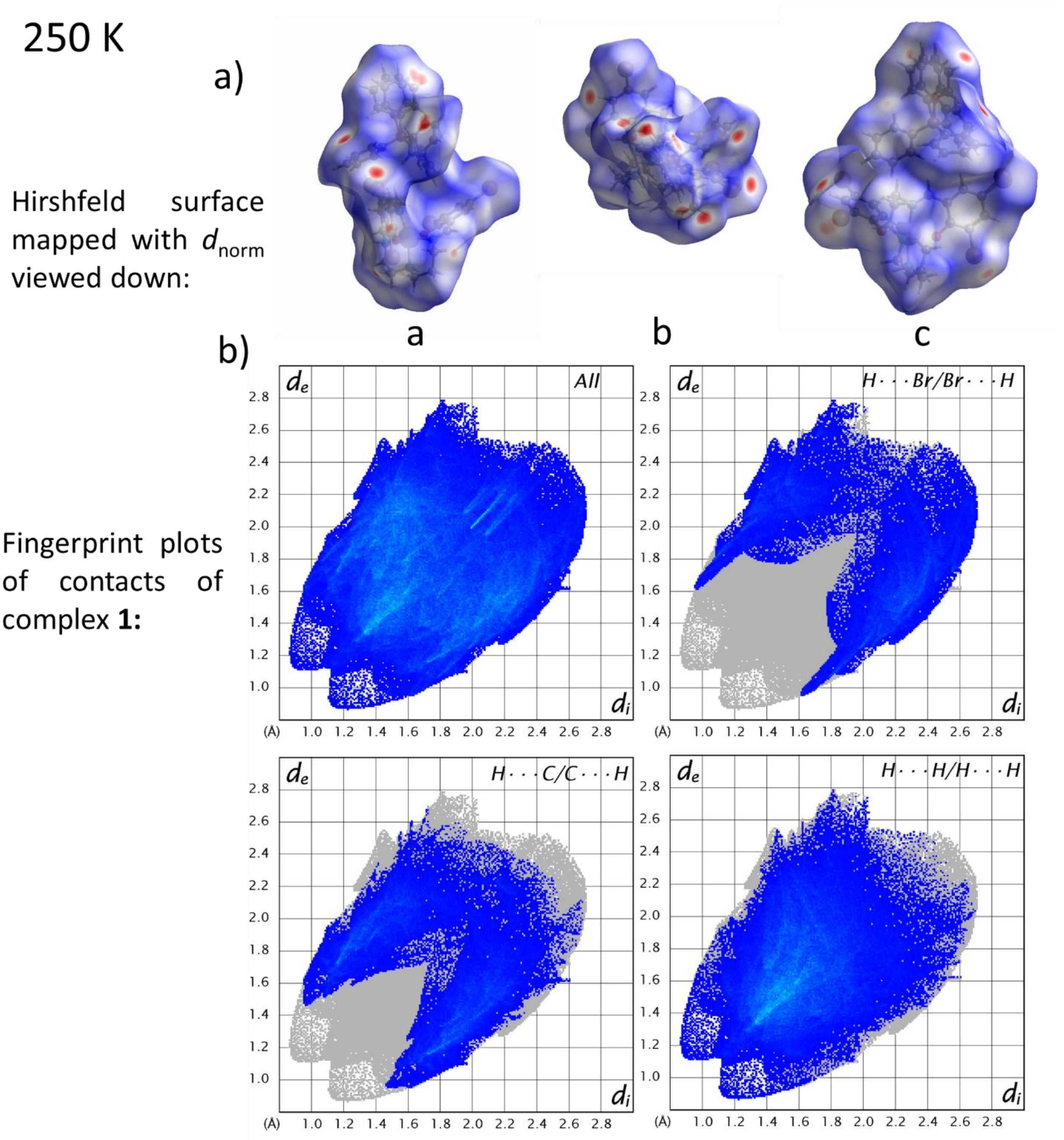

Figure S26: a) Hirshfeld surface mapped with d_{norm} for the structure measured at 250 K viewed along the *a*, *b* and *c* direction, b) fingerprint plots with all intermolecular interaction further resolved into the contribution of H···Br/Br···H, H···C/C···H and H···H/H···H contacts of complex **1** at 250 K.

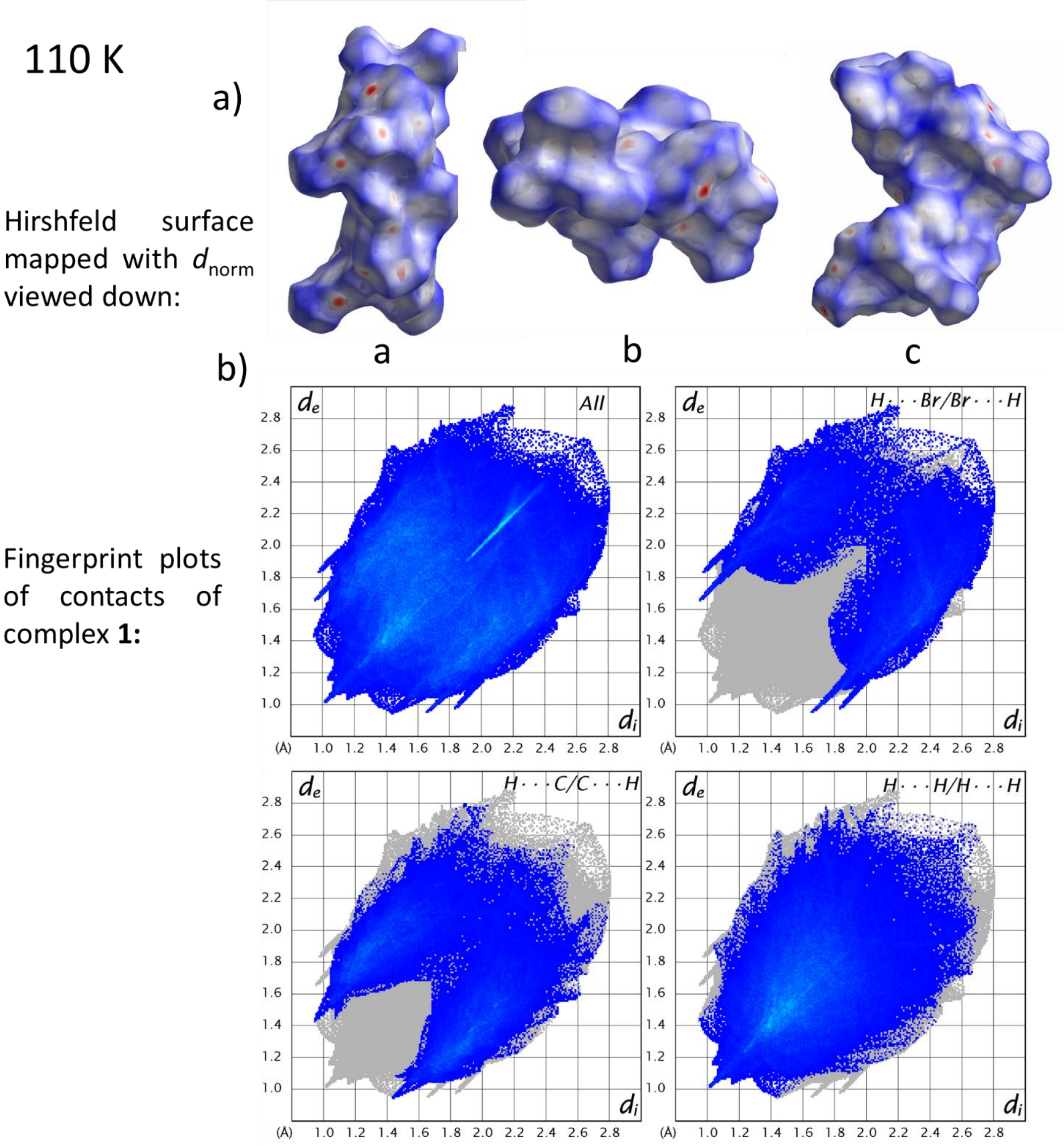

Figure S27: a) Hirshfeld surface mapped with d_{norm} for the structure measured at 110 K viewed along the *a*, *b* and *c* direction, b) fingerprint plots with all intermolecular interaction further resolved into the contribution of H···Br/Br···H, H···C/C···H and H···H/H···H contacts of complex **1** at 110 K.

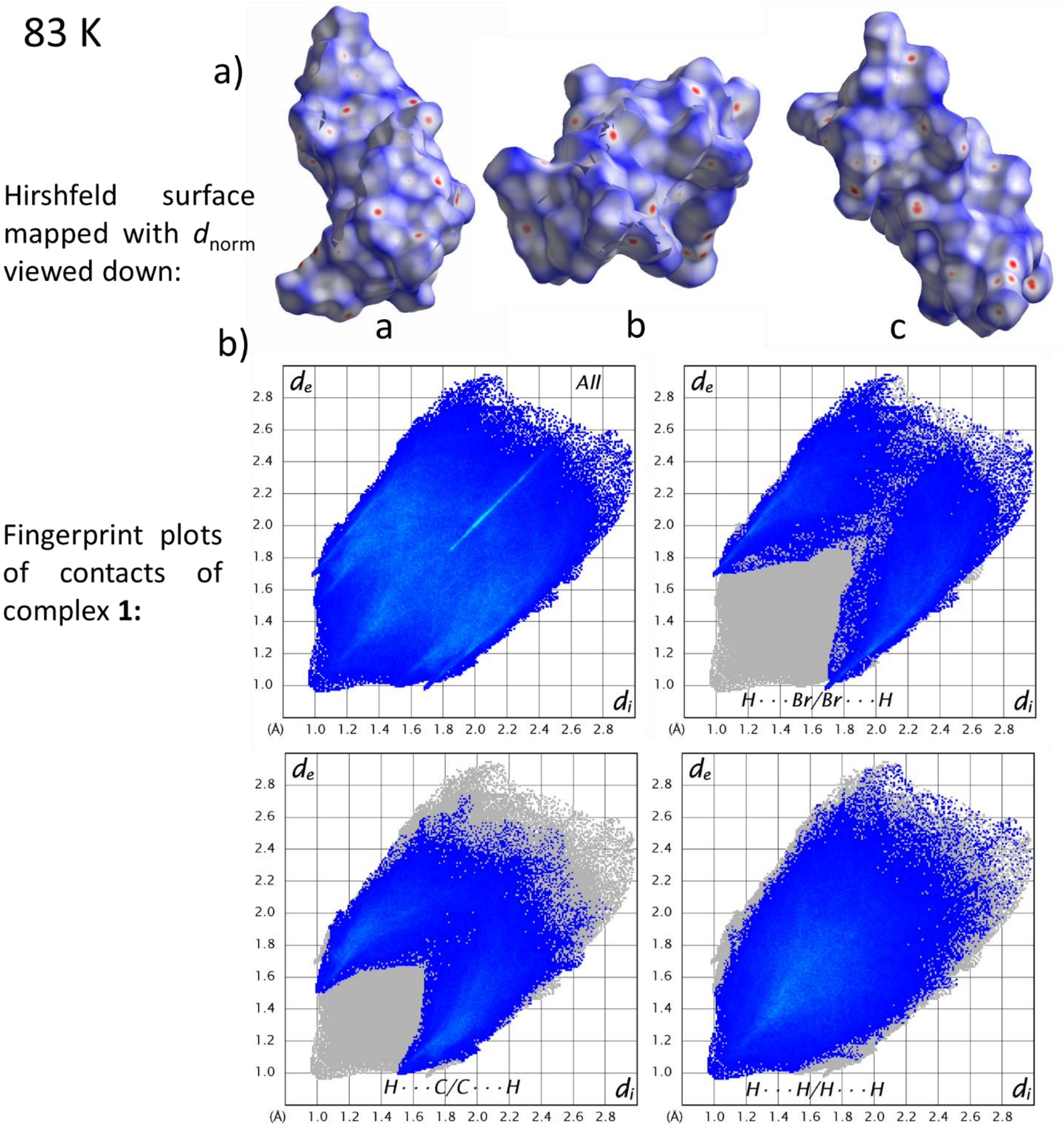

Figure S28: a) Hirshfeld surface mapped with d_{norm} for the structure measured at 83 K viewed along the *a*, *b* and *c* direction, b) fingerprint plots with all intermolecular interaction further resolved into the contribution of H···Br/Br···H, H···C/C···H and H···H/H···H contacts of complex **1** at 83 K.

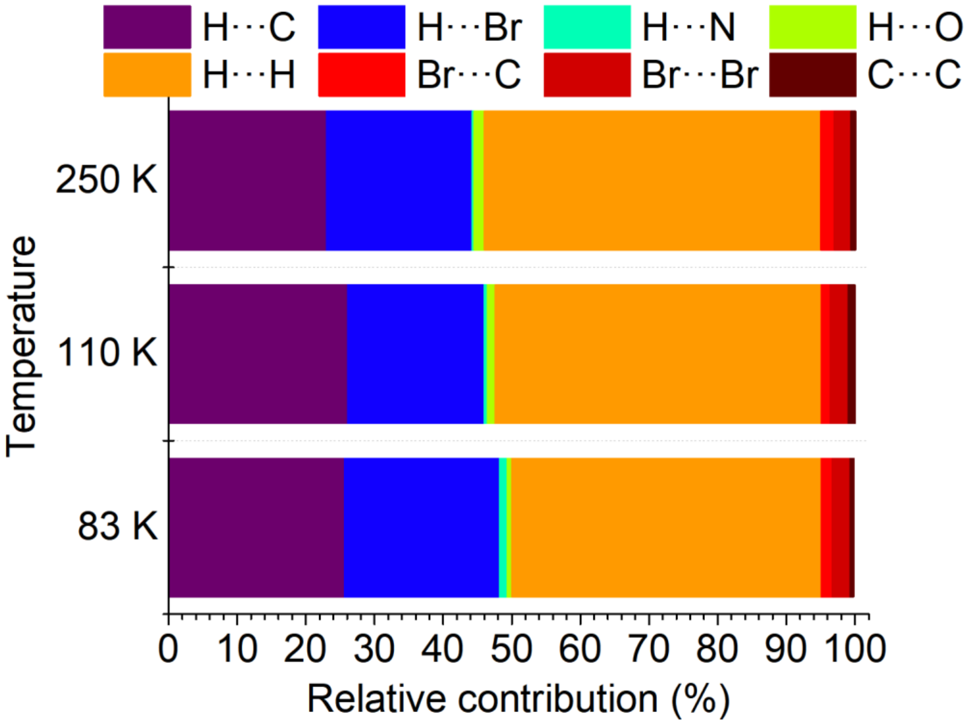

Figure S29: Relative contributions to the Hirshfeld surface area.of different intermolecular interactions for the HT (250 K), INT (110 K) and LT (83 K) structures.

The dominating interactions originates from H···C, H···Br, and H···H between the hydrogen atoms on the tetraphenylborate anion and the bromide groups on the phenyl ring on the salicylaldehyde motif as well as hydrogen and carbon atoms in the backbone of Mn^{3+} chelated complex. Those interactions appear as distinct spikes in the 2D fingerprint plot.

3.5 Variable temperature single crystal X-ray diffraction

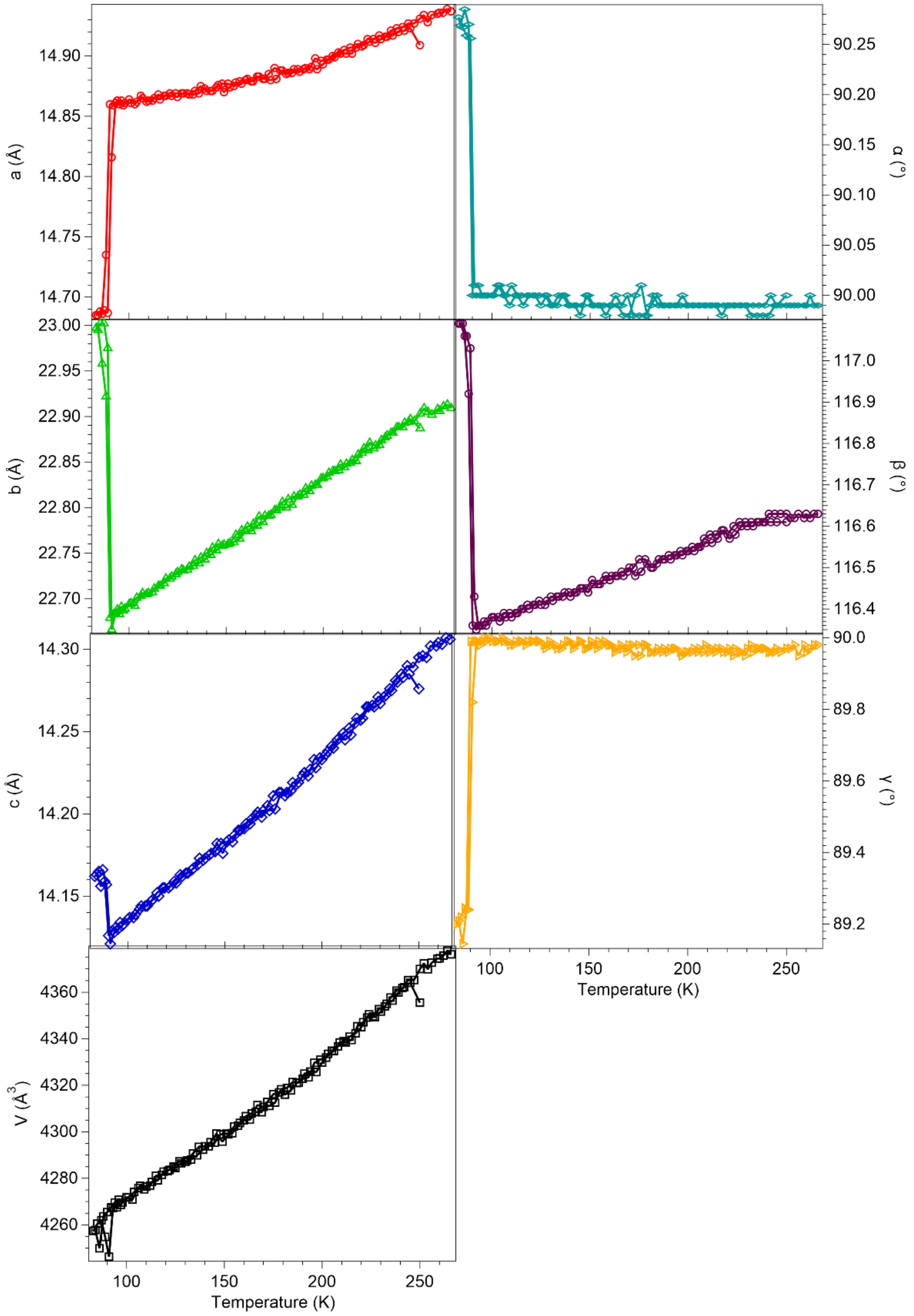

Figure S30: Variable temperature X-ray diffraction of unit cell parameters, *V, a, b, c, α, β* and *γ*, measured on a single crystal of complex **1** showing the cooling and heating sequence and changes in unit cell parameters from 83-266 K.

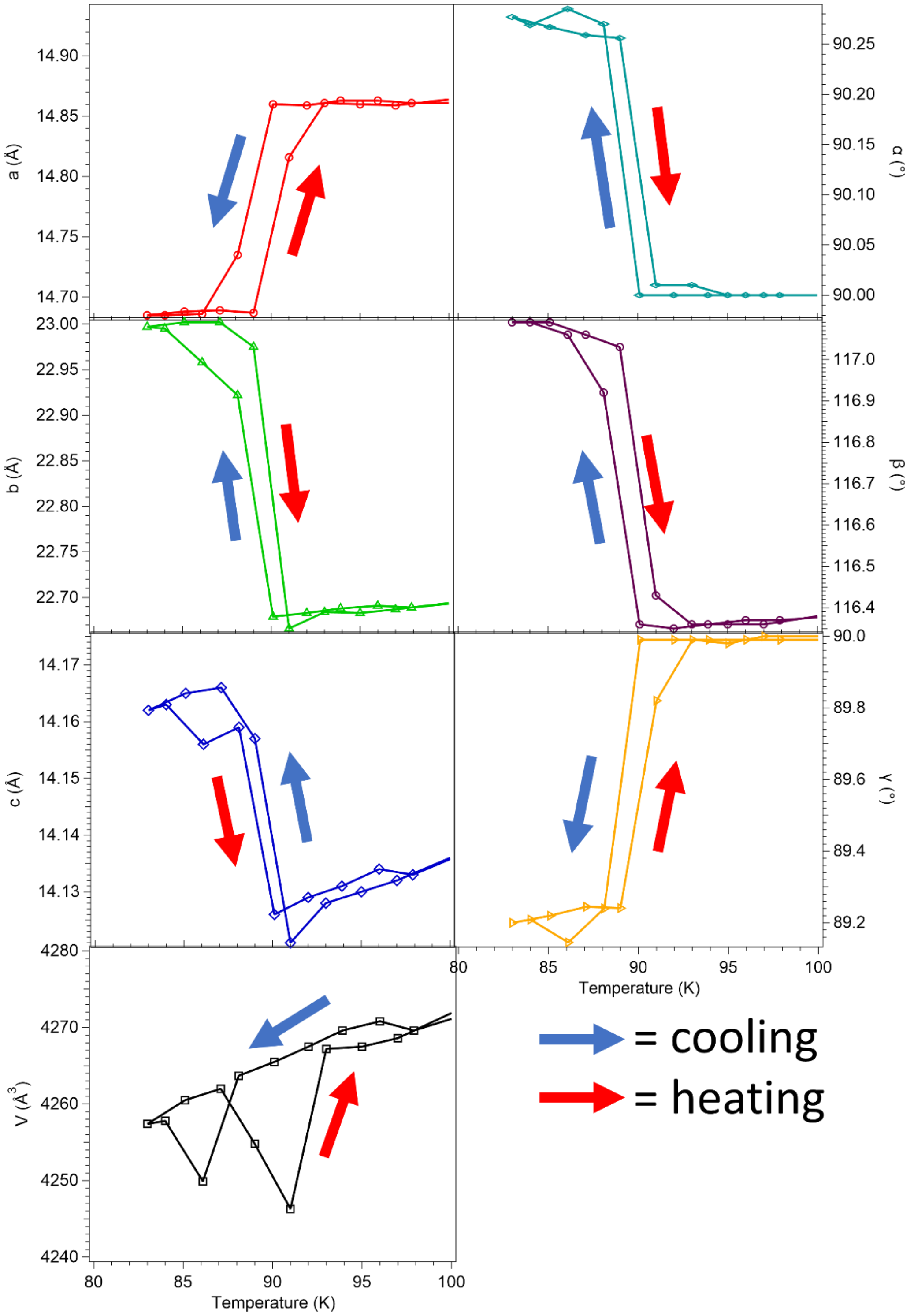

Figure S31: Variable temperature X-ray diffraction of unit cell parameters, *V*, *a, b, c, α, β* and *γ*, measured on a single crystal of complex **1** showing the cooling and heating sequence and changes in unit cell parameters from 80-100 K with arrows indicating the heating (red) and cooling mode (blue) of the data points.

The change in lattice parameters versus temperature, Figure S31 and S32 were measured from 250 K to 84 K in cooling mode and 83 K to 266 K in heating mode. The measurements were performed in 3 K per step from 250 to 101 K and in 2 K per step from 98 K to 84 K in cooling mode. In heating mode, the measurements were performed in 2 K per step from 83 K to 97 K and in 3 K per step from 100 K to 266 K. The data was integrated with respect to the *Cc* cell at 266 K.

3.6 Reconstructed planes and superstructure reflections

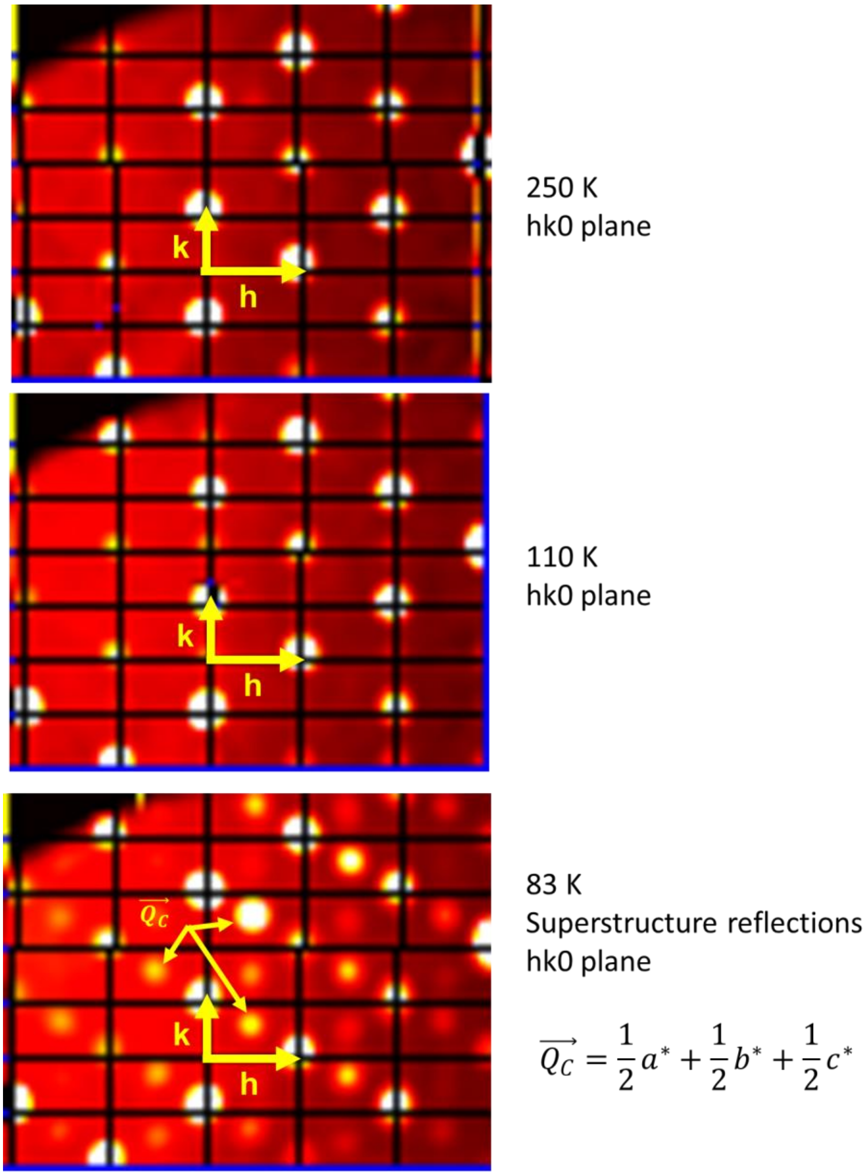

Figure S32: Representative precession images (hk0 plane) obtained from SCXRD of complex **1** collected at 250 K, 110 K and 83 K.
.

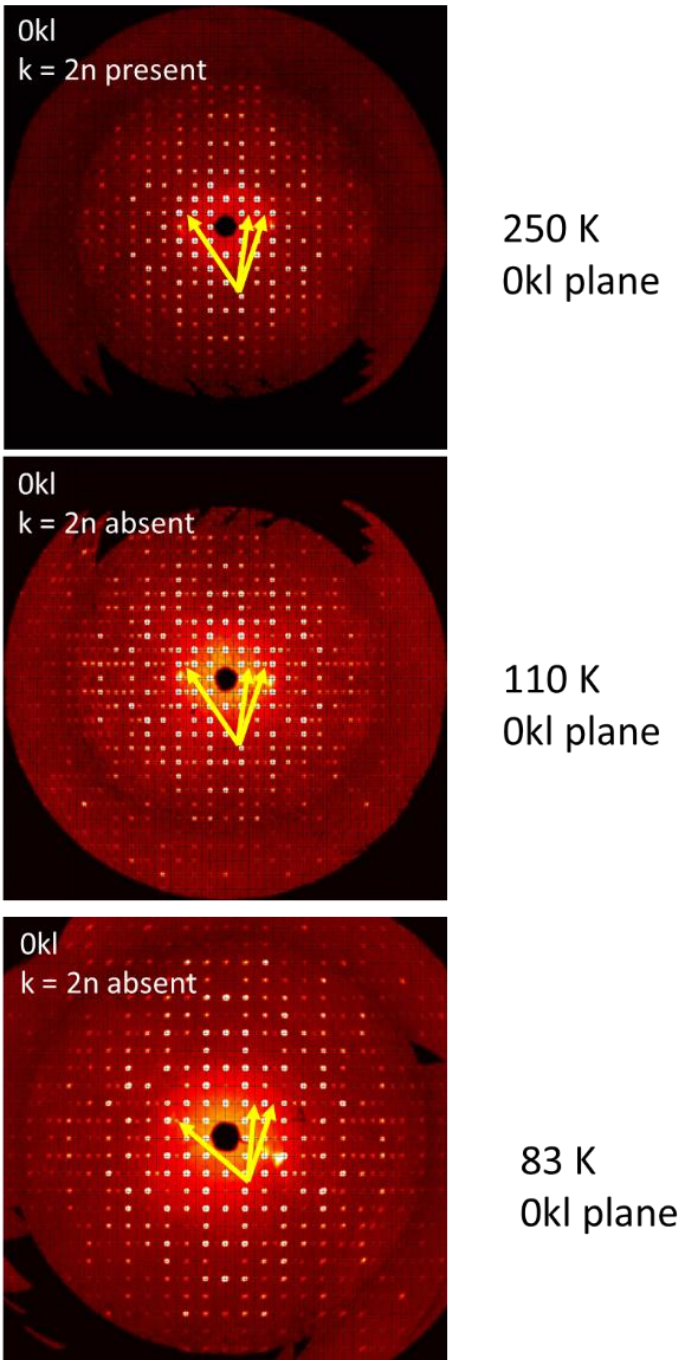

Figure S33: Representative precession images (0kl plane) was obtained from SCXRD of complex **1** collected at 250 K, 110 K and 83 K.

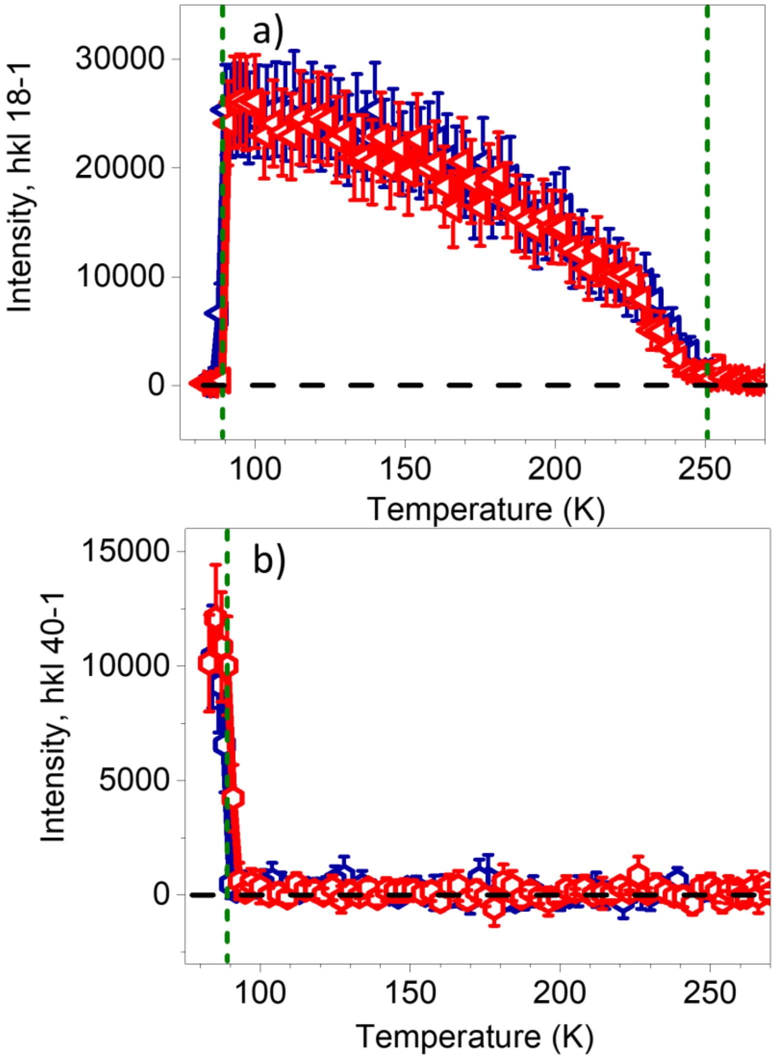

Figure S34: Appearance of superstructure reflections with error bars going from *Cc* → *Pc* for hkl = 18-1 and *Pc* → *P1* for hkl = 40-1.

3.7 Twin domains

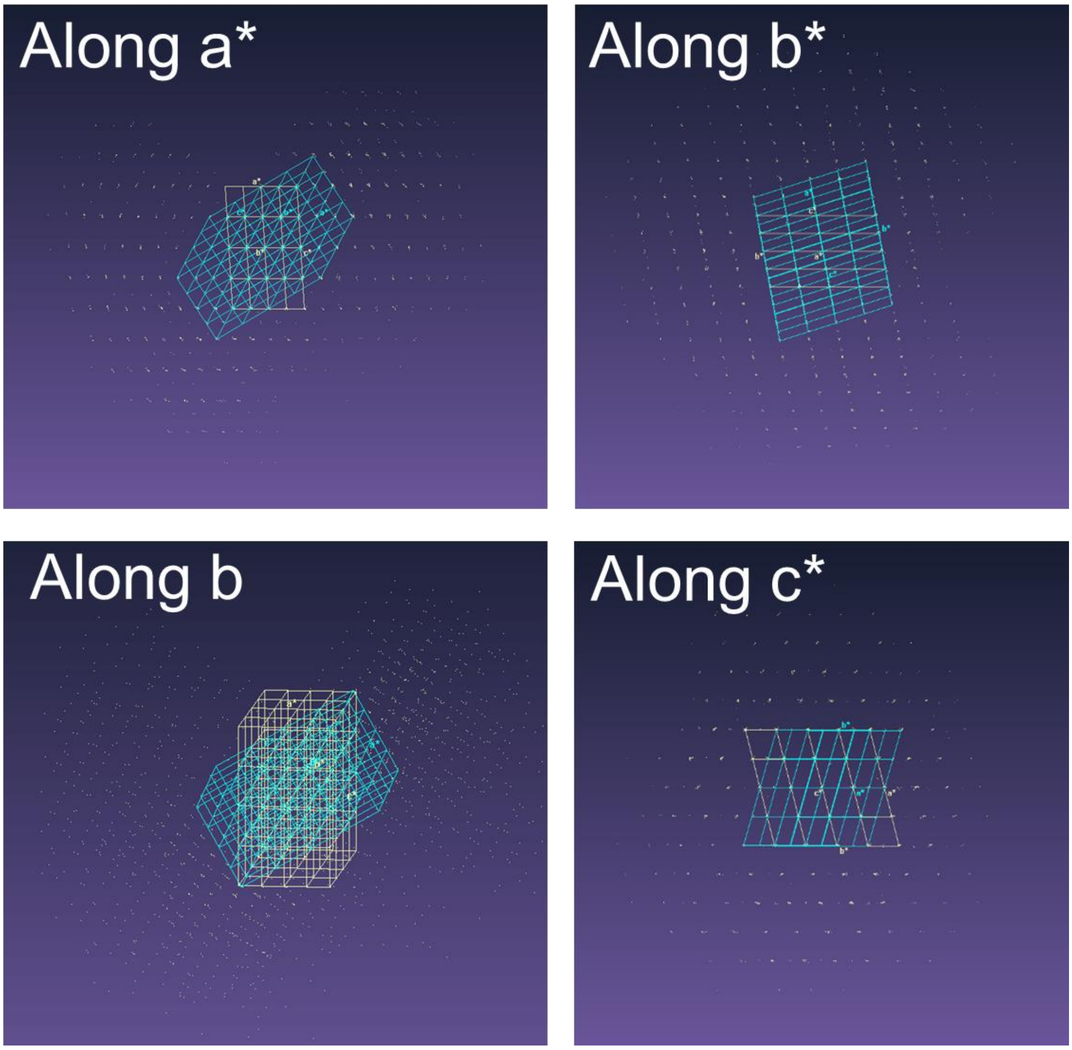

Figure S35: View of reciprocal space in various orientations of the unit cell at 83 K where presence of a second component illustrates the twin domain formed in the *P1* structure.

4. Resonant ultrasound spectroscopy

4.1 Strain analysis from unit cell parameters

Spontaneous strains associated with the two structural phase transitions have been determined using the approach and equations as previously set out.[10] The parent structure has space group *Cc* and the two derivative structures have space groups *Pc* and *P1*, with group-subgroup sequences as *Cc* → *Pc* (co-elastic) and *Cc* → *P1* (improper ferroelastic). Values of reference parameters a_o, b_o, c_o, α_o (=90°), β_o^* (= 180 - β_o), γ_o (=90°) for the *Cc* structure were obtained by extrapolation of a linear fit to measured parameters in the temperature interval 266 K to 250 K, Figure S36. A linear extrapolation does not allow for the requirement that the slope of lattice parameters of crystalline materials must tend to zero as $T \rightarrow 0$, but there are insufficient data for the *Cc* structure to allow fitting with a coth function as would normally be used for analysis of strains at low temperatures.[11]

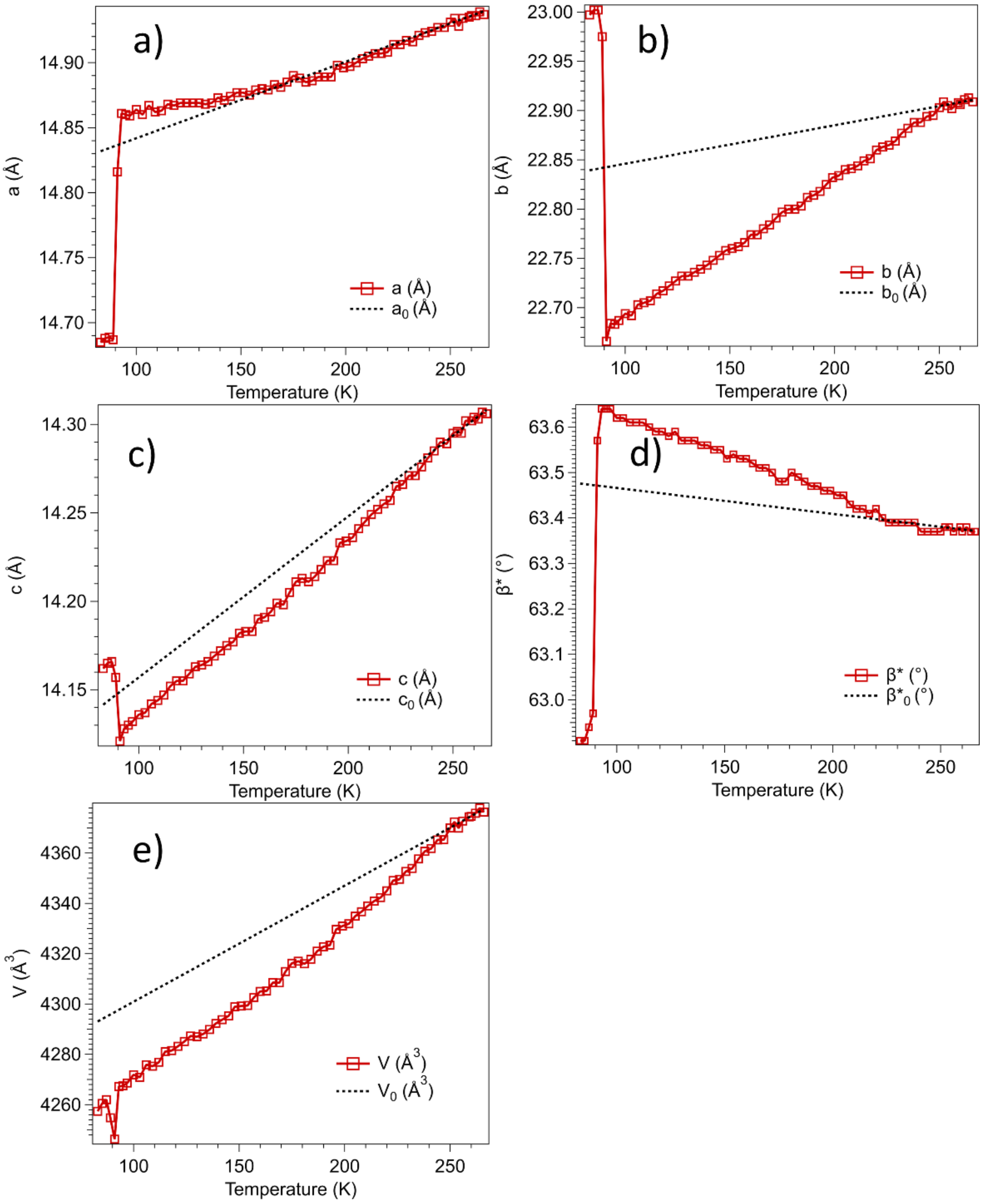

Figure S36: Linear fits to lattice parameter data in the temperature interval 266 to 83 K and the extrapolations to lower temperatures (dotted lines) represent variations of reference parameters a_o, b_o, c_o, α_o (=90°), β_o, γ_o (=90°) in calculations of the spontaneous strains associated with *Cc* → *Pc* and *Cc* → *P1* transitions. Reference values of the unit cell volume, V_o, were obtained in the same way.

Individual strain components, e_i, i = 1, 2, 3, 5 were calculated for *Cc* → *Pc* ($e_4 = e_6 = 0$) according to the following equations:

$$e_1 = \frac{a - a_0}{a_0} \quad (S1)$$

$$e_2 = \frac{b - b_0}{b_0} \quad (S2)$$

$$e_3 = \frac{c\sin\beta - c_0\sin\beta_0}{c_0\sin\beta_0} \quad (S3)$$

$$e_5 = \left(\frac{c\cos\beta}{c_0\sin\beta_0} - \frac{a\cos\beta_0}{a_0\sin\beta_0}\right) \quad (S4)$$

Individual strain components, e_i, i = 1- 6 were calculated for *Cc* → *P1* according to

$$e_1 = \frac{a}{a_0}\sin(\gamma) - 1 \quad (S5)$$

$$e_2 = \frac{b}{b_0} - 1 \quad (S6)$$

$$e_3 = \frac{c\sin(\alpha)\sin(\beta^*)}{c_0\sin(\beta_0^*)} - 1 \quad (S7)$$

$$e_4 = \left(\frac{c\cos(\alpha)}{c_0\sin(\beta_0^*)} + \frac{a\cos(\beta_0^*)\cos(\gamma)}{a_0\sin(\beta_0^*)}\right) \quad (S8)$$

$$e_5 = \left(\frac{a\sin(\gamma)\cos(\beta_0^*)}{a_0\sin(\beta_0^*)} + \frac{c\,sin(\alpha)\cos(\beta^*)}{c_0\sin(\beta_0^*)}\right) \quad (S9)$$

$$e_6 = \left(\frac{a}{a_0}\cos(\gamma)\right) \quad (S10)$$

Values of the volume strain, V_s, were given by

$$V_s = \frac{V - V_0}{V_0} \quad (S11)$$

The resulting strain variations are given in Figures 3a and 3b of the main text. A test of the accuracy of linear extrapolations to obtain the reference parameters is provided by comparison of values for V_s obtained directly using Equation S11 with values obtained using $V_s = e_1 + e_2 + e_3$, which should hold for small volume strains. As shown in Figure 3a, the two variations of V_s have the same non-linear form of temperature dependence but with different absolute values. It is safe to conclude that the strain components all have a continuous variation through the *Cc* → *Pc* transition point and a non-linear dependence on temperature in the stability field of the *Pc* structure. Errors in the absolute values must increase with falling temperature, however, because of the assumption of linearity for the reference parameters. The *Pc* → *P1* transition is clearly discontinuous, as expected given that two space groups do not have a group-subgroup relationship. In principle it should be possible to determine the transition temperature for the *Cc* → *P1* transition by extrapolation of a fit to e_4 and e_6 to zero but there are insufficient data to produce a reliable result.

For each of the *Cc* → *Pc* and *Cc* → *P1* transitions, the lowest order terms for coupling between strains, e_i, and the driving order parameter, q, have the form $\lambda e_i q^2$. This leads to the expected relationships $e_i \propto V_s \propto q^2$.[10]

4.2 Correlation of transition points between different measurements

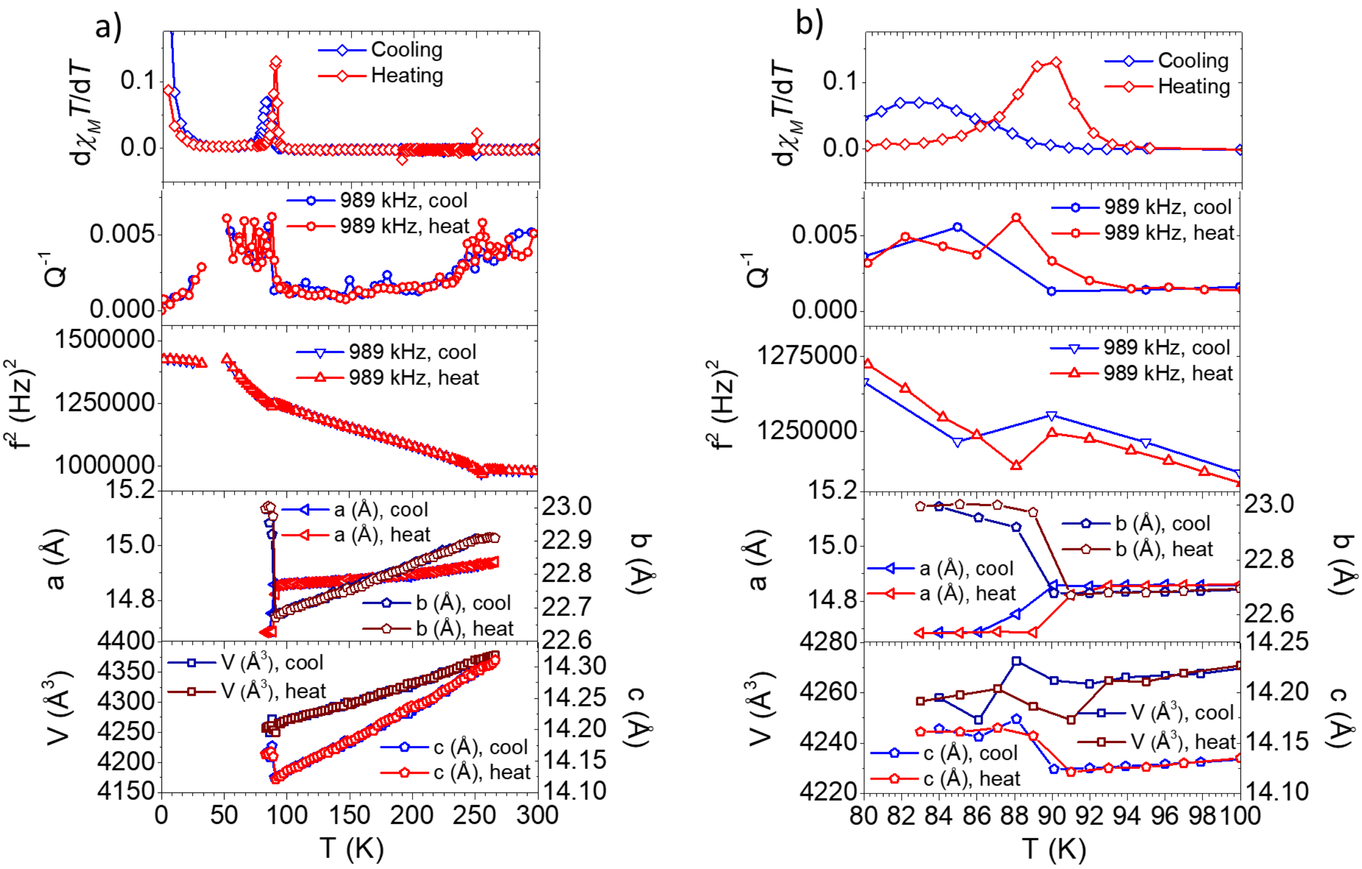

Figure S37: Correlation between heating and cooling modes of changes in d$\chi_M T/T$ vs T measured by SQUID magnetometry on a bulk polycrystalline sample, f^2 and Q^{-1} parameters elucidated from the RUS measurement on a single crystal and the variable temperature SCXRD of unit cell parameters measured on a single crystal shown in the range a) 0 K to 300 K and b) 80 K to 100 K.

5. Author contributions

V.B.J. and L.C.G. carried out the experimental procedures and characterization of samples. V.B.J. was supporting in data acquisition or analysis for all the experiments undertaken in this study. E.D., S.C., X.D. and V.S.Z. performed the PPMS data acquisition of the heat capacity and data analysis. K.E. and S.F. performed the SQUID magnetometry measurements. H.M.-B. and E.T. performed the single crystal X-ray diffraction experiments and data analysis of the single crystal X-ray data. M.A.C. collected the resonant ultrasound spectroscopy spectra and performed parts of the data analysis of RUS spectra and strain calculations. G.G.M. conceived and supervised the study. V.B.J., V.S.Z., M.A.C., E.C. and G.G.M. contributed to the main writing of the manuscript. All other authors made smaller contributions to the reading and writing of the manuscript.

6. References

[1] J. C. Lashley, M. F. Hundley, A. Migliori, J. L. Sarrao, P. G. Pagliuso, T. W. Darling, M. Jaime, J. C. Cooley, W. L. Hults, L. Morales, D. J. Thoma, J. L. Smith, J. Boerio-Goates, B. F. Woodfield, G. R. Stewart, R. A. Fisher, N. E. Phillips, *Cryogenics* **2003**, *43*, 369-378.

[2] CrysAlisPro v1.171.36-38, *Agilent Technologies XRD Products*, Oxfordshire.

[3] a) G. Sheldrick, *Acta Crystallogr. Sect. A: Found. Crystallogr.* **2008**, *64*, 112-122; b) G. Sheldrick, *Acta Crystallogr. Sect. A: Found. Crystallogr.* **2015**, *71*, 3-8.

[4] G. Sheldrick, *Acta Crystallogr. Sect. C: Cryst. Struct. Commun.* **2015**, *71*, 3-8.

[5] O. V. Dolomanov, L. J. Bourhis, R. J. Gildea, J. A. K. Howard, H. Puschmann, *J. Appl. Crystallogr.* **2009**, *42*, 339-341.

[6] R. E. A. McKnight, M. A. Carpenter, T. W. Darling, A. Buckley, P. A. Taylor, *Am. Mineral.* **2007**, *92*, 1665-1672.

[7] R. Ketkaew, Y. Tantirungrotechai, D. J. Harding, P. Harding, M. Marchivie, OctaDist: A tool for calculating distortion parameters in coordination complexes. https://octadist.github.io.

[8] H. Flack, *Acta Crystallogr. Sect. A: Found. Crystallogr.* **1983**, *39*, 876-881.

[9] M. J. Turner, J. J. McKinnon, S. K. Wolff, D. J. Grimwood, P. R. Spackman, D. Jayatilaka, M. A. Spackman, CrystalExplorer17, **2017**, University of Western Australia.

[10] M. A. Carpenter, E. K. H. Salje, A. Graeme-Barber, *Eur. J. Mineral.* **1998**, *10*, 621-691.

[11] a) E. K. H. Salje, B. Wruck, H. Thomas, *Z. Phys. B: Condens. Matter* **1991**, *82*, 399-404; b) M. A. Carpenter, H.-W. Meyer, P. Sondergeld, S. Marion, K. S. Knight, *Am. Mineral.* **2003**, *88*, 534-546.
